# Structures of the cGMP-dependent protein kinase in malaria parasites reveal a unique structural relay mechanism for activation

**DOI:** 10.1073/pnas.1905558116

**Published:** 2019-06-25

**Authors:** Majida El Bakkouri, Imène Kouidmi, Amy K. Wernimont, Mehrnaz Amani, Ashley Hutchinson, Peter Loppnau, Jeong Joo Kim, Christian Flueck, John R. Walker, Alma Seitova, Guillermo Senisterra, Yoshito Kakihara, Choel Kim, Michael J. Blackman, Charles Calmettes, David A. Baker, Raymond Hui

**Affiliations:** ^a^Structural Genomics Consortium, University of Toronto, Toronto, ON M5G 1L7, Canada;; ^b^INRS-Institut Armand-Frappier, Laval, QC H7V 1B7, Canada;; ^c^Department of Pharmacology, Baylor College of Medicine, Houston, TX 77030;; ^d^Faculty of Infectious and Tropical Diseases, London School of Hygiene & Tropical Medicine, London WC1E 7HT, United Kingdom;; ^e^Department of Biochemistry, University of Toronto, Toronto, ON M5S 1A1, Canada;; ^f^Verna and Marss McLean Department of Biochemistry and Molecular Biology, Baylor School of Medicine, Houston, TX 77030;; ^g^Malaria Biochemistry Laboratory, The Francis Crick Institute, London NW1 1AT, United Kingdom;; ^h^Toronto General Hospital Research Institute, Toronto, ON M5G 2C4, Canada

**Keywords:** cyclic GMP, signal transduction, malaria, *Plasmodium*, structure

## Abstract

The cyclic guanosine-3′,5′-monophosphate (cGMP)-dependent protein kinase (PKG) was identified >25 y ago; however, efforts to obtain a structure of the entire PKG enzyme or catalytic domain from any species have failed. In malaria parasites, cooperative activation of PKG triggers crucial developmental transitions throughout the complex life cycle. We have determined the cGMP-free crystallographic structures of PKG from *Plasmodium falciparum* and *Plasmodium vivax*, revealing how key structural components, including an N-terminal autoinhibitory segment (AIS), four predicted cyclic nucleotide-binding domains (CNBs), and a kinase domain (KD), are arranged when the enzyme is inactive. The four CNBs and the KD are in a pentagonal configuration, with the AIS docked in the substrate site of the KD in a swapped-domain dimeric arrangement. We show that although the protein is predominantly a monomer (the dimer is unlikely to be representative of the physiological form), the binding of the AIS is necessary to keep *Plasmodium* PKG inactive. A major feature is a helix serving the dual role of the N-terminal helix of the KD as well as the capping helix of the neighboring CNB. A network of connecting helices between neighboring CNBs contributes to maintaining the kinase in its inactive conformation. We propose a scheme in which cooperative binding of cGMP, beginning at the CNB closest to the KD, transmits conformational changes around the pentagonal molecule in a structural relay mechanism, enabling PKG to orchestrate rapid, highly regulated developmental switches in response to dynamic modulation of cGMP levels in the parasite.

## Significance

Despite being one of the first protein kinases discovered, cyclic guanosine-3′,5′-monophosphate (cGMP)-dependent protein kinase (PKG) has not been successfully crystallized previously, leaving many unanswered questions about its mechanism of activation. We report herein the structure of cGMP-free PKG from *Plasmodium* parasites, the causative agents of malaria, one of the world’s deadliest infectious diseases. The structures, combined with data from biochemical and biophysical experiments, provide insight into a mechanism of activation that involves previously unpredicted interdomain communication via a structural relay system. In addition to the full structure of PKG, our work contributes important functional information for a key antimalarial drug target.

Malaria remains a serious global health problem, with close to 500,000 deaths and hundreds of millions of new infections annually. Reports of prolonged parasite clearance times and treatment failures using artemisinin combination therapies (ACTs) are increasingly frequent in parts of Southeast Asia (1). New targets to supply the next generation of antimalarial drugs are being studied with urgency to tackle this growing trend, particularly in anticipation of the spread of ACT resistance to Africa. Among promising drug targets are protein kinases encoded by the genomes of the *Plasmodium* parasites responsible for the disease (2). Previous work has demonstrated that one particular kinase, known as cyclic guanosine-3′,5′-monophosphate (cGMP)-dependent protein kinase, or protein kinase G (PKG), has essential roles in multiple stages of the parasite life cycle. Selective pharmacological inhibition of *Plasmodium falciparum* PKG (*Pf*PKG) blocks the egress of merozoites (3) and gametes (4) from erythrocytes, as well as inhibits the motility of ookinetes in the mosquito (4, 5) and invasion of hepatocytes by sporozoites (6). PKG orchestrates the progression of these key differentiation events in *Plasmodium* via a complex system of second messenger signaling, involving phosphoinositide metabolism and calcium mobilization (7, 8). A phosphoproteomic study has linked *Pf*PKG activity to 107 phospho-sites on 69 different proteins in the *P. falciparum* proteome, including some implicated in invasion and egress (9). Regulation of cGMP has also been associated with calcium flux and egress in the related apicomplexan pathogens *Toxoplasma* and *Eimeria* (10). These lines of evidence support PKG as a promising target for antiparasitic drug discovery, as well as a gateway to a deeper understanding of parasite signaling.

The cellular functions of parasite PKG are regulated by allosteric and cooperative binding of cGMP (11), similarly to how mammalian PKG is activated. Allostery and cooperativity are also the hallmarks of another eponymous member of the AGC subfamily of protein kinases, adenosine 3′,5′-cyclic monophosphate (cAMP)-dependent protein kinase (PKA). The regulatory and catalytic subunits of PKA are distinct, separately encoded proteins that form tetrameric complexes composed of two regulatory subunits and two catalytic subunits (R2C2) (12, 13). In contrast, the regulatory and catalytic domains of PKG coexist within a single polypeptide encoded by a single gene. In mammals, this assembly contains two cyclic nucleotide-binding (CNB) sites, compared with the four CNB domains in apicomplexan PKG isoforms (14). Even though PKA and PKG were both among the earliest kinases identified, understanding of how the structural divergence of PKG from PKA affects cGMP-mediated allostery and cooperativity (15) in different organisms is at best fragmentary.

Structural biology is an essential tool in the study of protein kinases. PKA, the first to be crystallized (16), is the archetypal protein kinase in structural and mechanistic studies. There are now a number of PKA structures revealing the standalone catalytic and regulatory subunits, as well as the engaged R1C1 dimers and R2C2 tetramers (12, 17, 18). In contrast, for both mammalian and parasite PKG, only the structures of isolated regulatory domains have been reported (19–26). A report disclosing the structure of one of the cGMP-binding domains of *Pf*PKG (CNB-D) identified a triad of residues in its C-terminal helix that are essential for regulation of enzyme activity (23). How this domain interacts with the remainder of the PKG structure and, more generally, how *Plasmodium* PKG mediates cGMP signaling are unknown.

Here, we describe the complete atomic structure of PKG from two human malaria parasite species, *P. falciparum* and *Plasmodium vivax*. To date this has not been achieved for PKG from any other organism. The resulting structures provide insights into how the CNBs interact with the kinase domain (KD) to keep it in a largely autoinhibited state and how the parasite maintains its cGMP signaling system in the off state. Combining our structural findings with biophysical and biochemical data allows us to propose a structural relay model of cooperative activation of parasite PKG that may also have important implications for regulation of mammalian PKG.

## Results

### *Plasmodium* PKG Is a Member of a Distinct Class of PKG Enzymes.

**Fig. 1.**
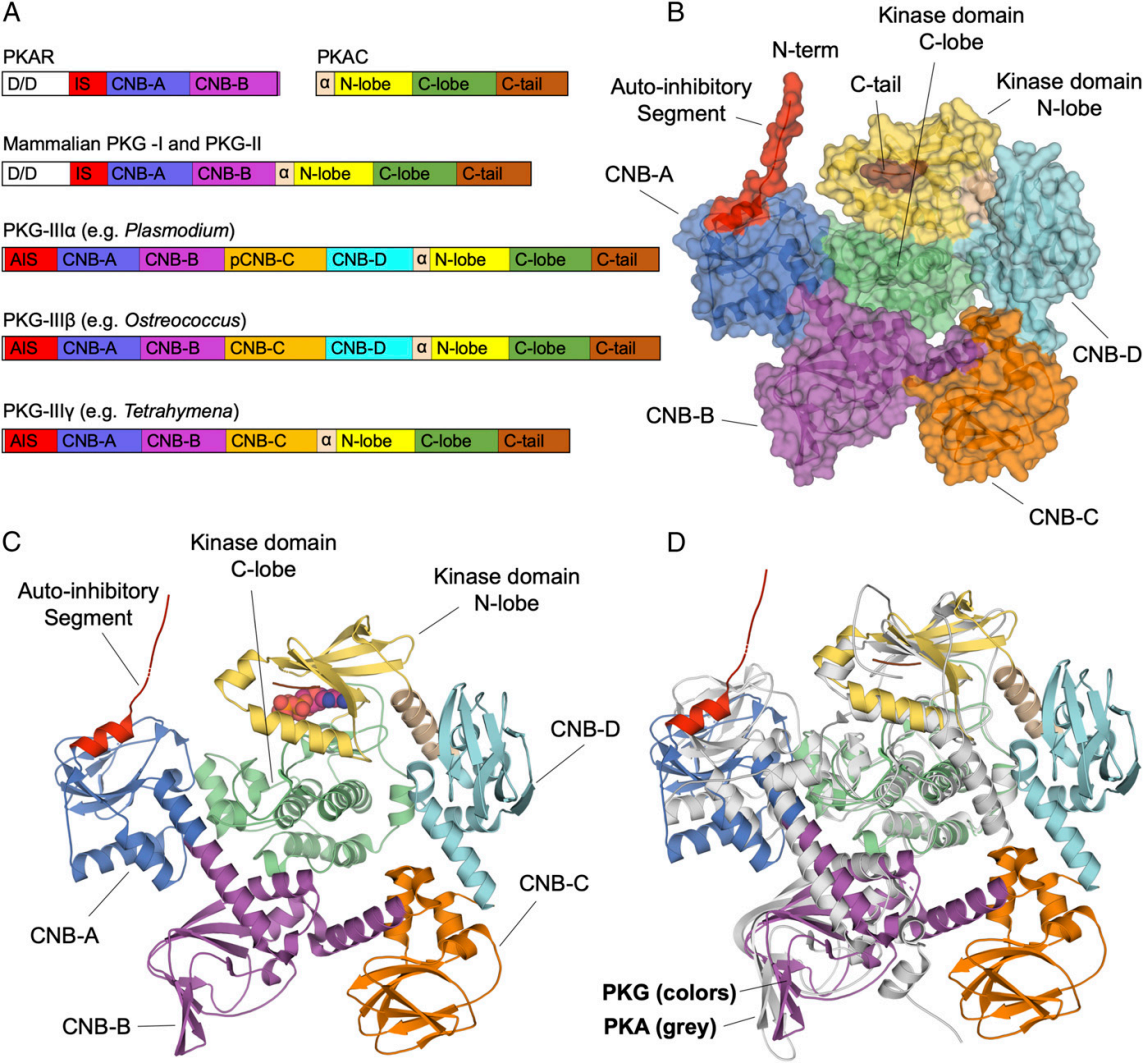
*Plasmodium* PKG displays a pentagonal architecture. (*A*) PKA is made up of two proteins: The regulatory subunit (R) contains CNB-A and -B and the catalytic subunit (C) includes an N-terminal helix (pink), the classical bilobal KD (yellow and green), and a C terminus (brown). Mammalian PKG-I and -II feature a PKA-like catalytic KD concatenated with a tandem of CNBs. In type III PKG, there are three or more CNB or CNB-like domains. *Pf*PKG, an exemplar of type IIIα, contains four integrated CNBs, one of which does not bind cGMP. In contrast, type IIIβ PKG has four functional cGMP-binding sites, while type IIIγ members have exactly three CNBs. An inhibitory substrate sequence flanks the N terminus of the first CNB in each case. In PKA and mammalian PKG, there is an additional region of dimerization and docking motifs. The absence of this in type III PKGs suggests that they are likely monomeric. (*B*) Surface rendering of *Pf*PKG colored as in *A*. *Pf*PKG adopts a pentagonal architecture composed of the four CNB domains and the N lobe KD in the outer rim, and the C lobe KD locked in the middle. The CNB-A and -B domains make contact with the C lobe of the KD, similar to what is seen in all PKA heterodimers. CNB-D also makes contact with the C terminus (brown), whereas pCNB-C does not make direct contact with any part of the KD. CNB-D shows a unique arrangement not seen in PKA, making contacts with both the N lobe and the C terminus. (*C*) Structure of *Pf*PKG drawn in cartoon representation. The long helix connecting CNB-A and -B is similar to that seen in PKA. We hypothesize that, inside the parasite, the AIS would occupy the KD’s active site to form a fully autoinhibited monomer. (*D*) Overlay of *Pf*PKG and PKA heterodimer (PDB ID code 2QCS; in gray) depicts structural homologies. CNB-A, -B, and the KD form a substructure that closely resembles the heterodimer of PKA.

Mammalian PKGs are classified into types Iα, Iβ, and II (also known as PKG-Iα, -Iβ, and -II), all featuring a single polypeptide comprising a regulatory domain of two CNBs fused to the N-terminal flank of a single catalytic KD. Known to form inactive dimeric holoenzymes when free of cGMP, they have an effective regulatory stoichiometry (i.e., a CNB-to-KD ratio) of 4:2, similar to that in a PKA R2C2 tetramer. In contrast, PKG enzymes of *Plasmodium* and other apicomplexan parasites have an extended N-terminal domain that features four CNBs (Fig. 1*A*) upstream of the KD and are monomeric (27), resulting in an effective regulatory stoichiometry of 4:1. There is thus significant structural divergence between *Plasmodium* and mammalian PKGs, prompting us to formally define kinases with more than two integrated cGMP-binding sites as type III or PKG-III.

A search of the National Center for Biotechnology Information (NCBI) Protein database (https://www.ncbi.nlm.nih.gov/protein) led us to identify multiple subclasses of type III PKGs. In all apicomplexan PKGs examined, one of the four CNBs is similar in sequence to canonical cGMP-binding domains but lacks one or more critical residues (23). We refer to such a domain as a pseudo-CNB (pCNB) and the corresponding PKG as type IIIα. There are organisms, including green algae such as *Ostreococcus*, in which PKG contains four cGMP-binding CNBs—that we call type PKG-IIIβ. Finally, type IIIγ PKGs, such as some found in *Paramecium* and *Tetrahymena* (both of which have multiple paralogues of PKG), contain three predicted CNBs. Type III PKGs are observed only in protist genomes. In contrast, animals possess only types I and II PKGs. The architectures of these subtypes, along with those for types I and II as well as PKA, are shown for comparison in Fig. 1*A*.

### *Plasmodium* PKG Adopts a Pentagon-Shaped Architecture.

**Fig. 2.**
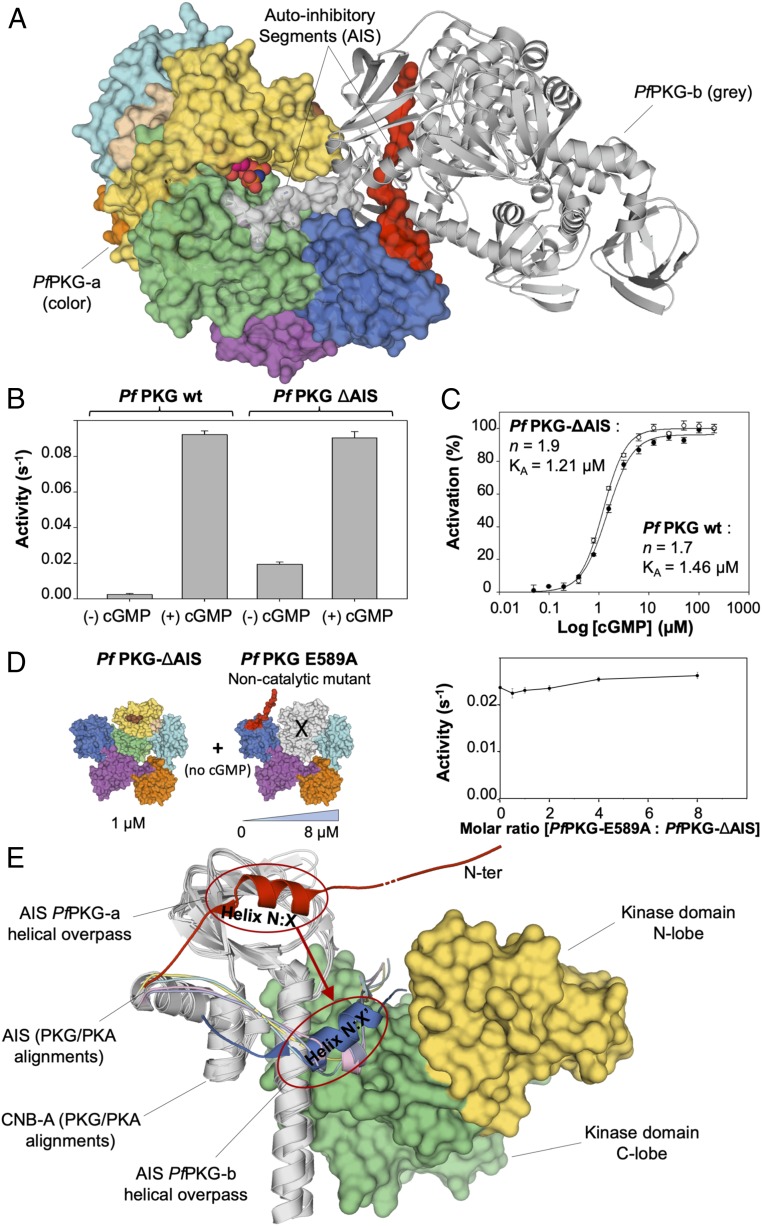
Biochemical and biophysical assays. (*A*) The representation of the asymmetric unit from the *Pf*PKG crystal illustrates a swapped domain dimer arrangement in which the N terminus of each protomer interacts with the active site of the other. The pseudosubstrate (red and gray surfaces) from the dimeric partner is shown binding (and inhibiting) the substrate site between the N and C lobes. These interactions contribute to autoinhibition of the protein. (*B*) The AIS inhibits PKG activity in vitro. In the absence of cGMP, the full-length protein exhibits negligible activity, whereas the truncated mutant protein (AIS removed) was partially active. Both samples were equally active in the presence of cGMP. wt, wild type. (*C*) Both the full-length and AIS-truncated constructs demonstrate homotropic positive cooperativity (Hill coefficient is 1.7 for the full-length sample and 1.9 for the truncated sample) when activated by addition of cGMP. The AIS is not required for full kinase activity under cGMP activation. (*D*) The AIS functions intrasterically to suppress kinase activity. The kinase activity of the catalytically active *Pf*PKG–ΔAIS truncation mutant (1 µM) is monitored in the absence of cGMP with incremental concentrations of full-length *Pf*PKG–E589A dead enzymes (1–8 µM). The AIS of the dead enzyme does not occupy/inhibit the active site of *Pf*PKG–ΔAIS. (*E*) Using the dimeric structure to hypothesize a model for the autoinhibitory domain of monomeric *Pf*PKG. The KD of one *Pf*PKG-a is shown in yellow and green surface, along with its adjacent CNB-A domain aligned with the CNB-A of all four types of PKA (PDB ID codes 3FHI, 2QVC, 4DIN, and 4X6Q; all drawn in gray). The AIS of *Pf*PKG-b (dark blue) overlays with the inhibitory segment of all four PKA structures (RIα in purple, RIIα in cyan, RIβ in pink, and RIIβ in yellow). In a monomer, we hypothesize that *Pf*PKG-a would extend its own AIS (colored in red) in the same position (instead of in the KD of *Pf*PKG-b). In all cases, a short helical overpass hovers above the long helix connecting the first two CNBs follows this inhibitory motif. The overpass of *Pf*PKG-b is shown here to indicate how *Pf*PKG-a might be different structurally as a monomer. It is also interesting that all of the structures are closely aligned from helix X:N onward, including *Plasmodium* PKG. This, along with all experimental data, supports the relevance of the rest of our crystal structure.

We expressed and purified full-length recombinant *Pf*PKG and *P. vivax* PKG (*Pv*PKG). They crystallized as dimers in orthorhombic and monoclinic space groups respectively, and both yielded 2.4-Å apo structures [Fig. 2*A* and *SI Appendix*, Fig. S1 and Table S1; Protein Data Bank (PDB) ID codes: 5DYK and 5DYL]. The two orthologs are 92% identical in sequence; furthermore, alignment of their structures resulted in rmsds of 0.8 Å—representing negligible differences (*SI Appendix*, Fig. S1). *Pv*PKG also cocrystallized with adenylyl-imidodiphosphate (AMPPNP), a nonhydrolyzable analog of adenosine triphosphate (ATP), again as a dimer (PDB ID code 5DZC; 2.3 Å; *SI Appendix*, Table S1). In the ensuing description, unless stated otherwise, important details are common to all of the structures. To ensure clarity, important differences in residues are mentioned in the text and highlighted in an alignment of the sequences of the two orthologs (*SI Appendix*, Fig. S2).

In the *Plasmodium* PKG crystal structures, each protomer in the dimers can be described as four CNBs, which, together with the N lobe of the KD, form a pentagonal arrangement (Fig. 1 *B* and *C*), with the KD C lobe locked in the center. CNB-A and -B make direct contact with and constrain (and are constrained by) the C lobe of the KD from functionally essential movement (Fig. 1 *B* and *C*), resulting in a substructure of these three domains that closely resembles the R-C heterodimer of PKA (Fig. 1*D*) (12). Interestingly, all three *Plasmodium* structures display a swapped domain arrangement, in which the N terminus of each protomer in the dimer interacts with the active site of the other (Fig. 2*A*).

### The N-Terminal Segment of *Plasmodium* PKG Has an Autoinhibitory Role.

To study the significance of occupation of the *Plasmodium* PKG active site by the N-terminal residues, we generated a truncated form of *Pf*PKG which starts at position S30, and so lacks the swapped N-terminal motif (Fig. 2*A*). Kinase activity assays (Fig. 2*B*) showed that the truncated recombinant enzyme (*Pf*PKG–ΔAIS) had equivalent activity to the full-length protein in the presence of cGMP; however, only the truncated form demonstrated activity in the absence of cGMP (Fig. 2*B*). This suggested an inhibitory role for the N-terminal segment of *Plasmodium* PKG that is likely similar to that performed by an equivalent motif in PKA (18) and human PKG (28). Accordingly, we validated that the *trans*-binding of the N-terminal motif does not contribute to inhibition of the kinase (Fig. 2*D*), suggesting an intrasteric mode of action as demonstrated by preincubation of the catalytically active N-terminally truncated form of *Pf*PKG (*Pf*PKG–ΔAIS) with an inactive full-length *Pf*PKG–E589A enzyme (ATP-binding-site mutant). Furthermore, the activity curve of *Pf*PKG–ΔAIS is sigmoidal, with a Hill coefficient of 1.9 (Fig. 2*C*), indicative of the same homotropic positive cooperativity reported for full-length PKG in *Plasmodium* and other type IIIα PKGs.

The dimeric crystal structure was unexpected and required investigation of its relevance, which was performed by using multiangle laser light scattering (full-length *Pf*PKG and *Pv*PKG; *SI Appendix*, Fig. S3), analytical ultracentrifugation (full-length *Pf*PKG and *Pv*PKG; full-length *Pf*PKG with AMPPNP; and *Pf*PKG–ΔAIS; *SI Appendix*, Fig. S4), and immunoblot analysis of epitope-tagged native *Pf*PKG from parasite lysates (*SI Appendix*, Fig. S5). In all cases, the dimeric fraction ranged from very little (e.g., the strongest dissociation constant was found to be ∼32 µM in area-under-the-curve experiments, indicative of a very small dimer fraction) to undetectable. Collectively, the activity assays and biophysical characterizations indicate that (*i*) cGMP is required for full activation of *Pf*PKG; (*ii*) the interaction between the N terminus of the protein and the active site is required for complete autoinhibition (but not for cooperativity), prompting us to name this region the autoinhibitory segment (AIS) for parasite PKG; and (*iii*) *Plasmodium* PKG is predominantly monomeric [consistent with what has been reported for the closely related *Eimeria* PKG (29)]—i.e., the swapped-domain dimer is unlikely to be representative of the physiological form of the inactive or active protein. To date, our attempts to crystallize the AIS-truncated form of *Plasmodium* PKG, the monomeric state of the full-length (or truncated) protein, or its cGMP-bound configuration have been unsuccessful.

### Deviations of the KD of *Plasmodium* PKG from the PKA Catalytic Subunit Have Functional Implications.

**Fig. 3.**
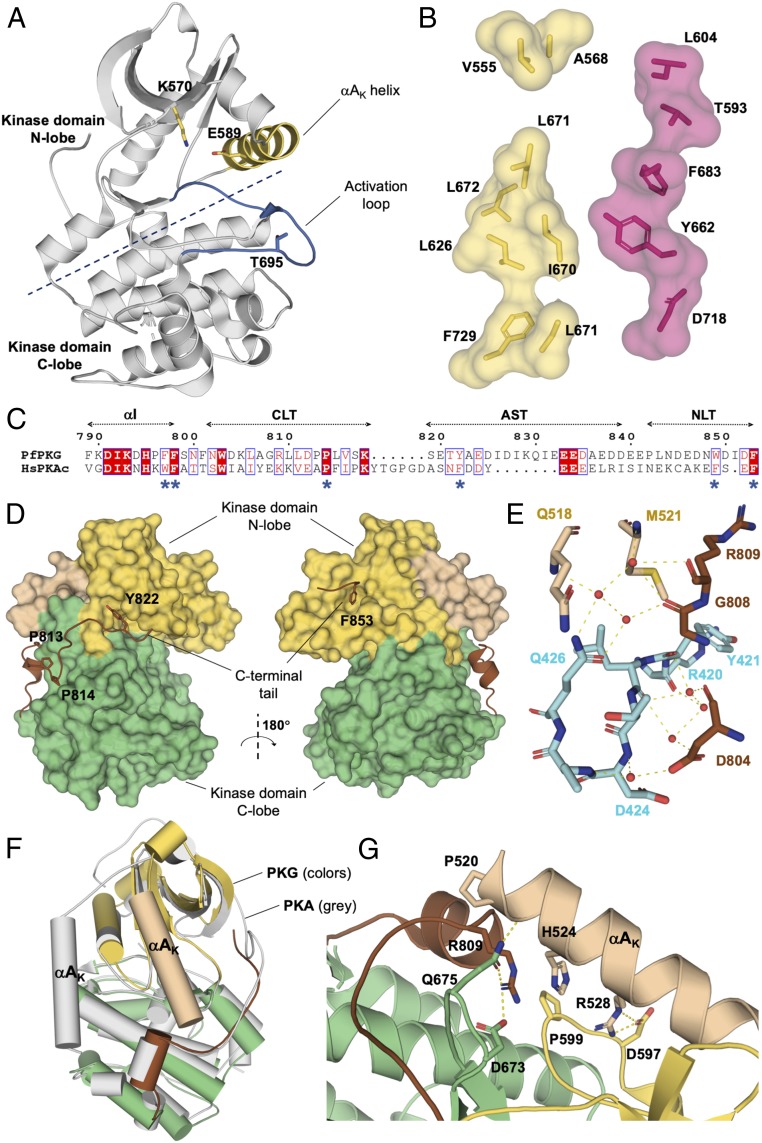
KD of *Pf*PKG. (*A*) KD of *Pf*PKG isolated with the rest of the protein hidden. The activation loop is highlighted in blue in part to show that it is in its active conformation, but does not contain a phosphorylated site. The residue T695 is unphosphorylated, which is unusual for an active AGC kinase structure. The interaction between K570 and E589, the position of the helix αA_K_, and the conformation of the activation loop are all characteristic of an active KD. (*B*) Intact hydrophobic spines are characteristic of an active KD. The C spine, shown here in yellow, comprises V555, A568, L626, I670, L671, L672, C725, and F729. It is broken in two sections because this crystal structure is missing ATP or a similar ligand. The R spine, made up of T593, L604, Y662, F683, and D718, is in red. (*C*) Similar to human PKA, the C terminus of *Pf*PKG comprises helix αI, the CLT, the AST, and the NLT. (*D*) Two prolines (P813 and P814) anchor *Pf*PKG’s CLT in a similar fashion as the PxxP (P314, F315, and P317) motif does in PKA. The FDDY motif in PKA is replaced by a single tyrosine (Y822) in *Pf*PKG. The NLT in *Pf*PKG comprises the motif WDIDF. In the structure, W849 is disordered, but F853 inserts itself in a hydrophobic pocket at the top of the N lobe of the KD. (*E*) The C tail of the KD (residues in brown) makes water-mediated contacts with CNB-D (cyan). (*F*) The main structural deviation between PKA and PKG is the N-terminal helix, as seen in the superposition of the catalytic subunit of PKA from mouse (PDB ID code 1ATP; αA_K_ in gray) and the KD of *Pf*PKG (αA_K_ in beige). (*G*) In *Pf*PKG-KD, helix αA_K_ interacts with both lobes of the KD as well as the C terminus, with a number of interactions maintaining the KD in an open conformation and the overall protein in its autoinhibited state. These interactions include a π-bond between H524 on helix αA_K_ and R809 on the C tail, with the latter secured by salt bridges with P599 in the N lobe and D673 and D675 in the C-lobe.

Similar to PKA, the KD of *Pf*PKG adopts a classical bilobal structure flanked by an N-terminal helix (αA_K_) and a C terminus (Fig. 3*A*). Despite being locked in an overall autoinhibited state, *Pf*PKG–KD surprisingly exhibits many structural features of an active kinase, including: (*i*) interaction between the catalytic lysine K570 and E589 (on helix αC_K_; Fig. 3*A*); (*ii*) positioning of the helix αC_K_ toward the ATP-binding site (Fig. 3*A*); (*iii*) an open (active) conformation of the activation loop (Fig. 3*A*); and (*iv*) intact hydrophobic spines (Fig. 3*B*). We further note that, compared with fully closed KDs (e.g., PDB ID code 1ATP), the KD in all our structures can be described as partially open, with the AMPPNP-bound structure less open than the nucleotide-free versions.

All three of our *Plasmodium* PKG structures feature an activation loop that is both in its open conformation and unphosphorylated—an uncommon combination previously observed in rho kinases and rho-associated kinases (30) such as ROCK and MRCK. This is in contrast to PKA (and other members of the AGC subfamily and the majority of available S/T kinases crystallized with the activation loop in the open position), where T197 is phosphorylated (16). In recombinant *Pf*PKG, we found that T695 (homologous to T197 in PKA) would become phosphorylated in the presence of cGMP, Mg^2+^, and ATP. The same modification has also been identified in a phosphoproteomics study (31). On the other hand, we found that the recombinant protein maintained the same activity profile (i.e., inactive without cGMP and fully active with cGMP) when T695 was mutated to either Ala or Gln (*SI Appendix*, Table S2). Rationalizing the significance of this putative phosphothreonine requires additional experiments.

The C terminus is a hallmark of AGC kinases (32) and an essential *cis*-regulatory component. It is partially disordered in our *Pf*PKG structure, similar to apo PKA structures (e.g., PDB ID code 1CTP). It is made up of four segments (Fig. 3*C*)—helix αI, the C-lobe tether (CLT), the active-site tether (AST), and the N-lobe tether (NLT). In the CLT, the first proline in the PxxP motif in PKA (and PKC)—known to play a role in interlobe movement—is conserved in *Pf*PKG (Fig. 3 *C* and *D*). In the AST, *Pf*PKG uses a motif starting with tyrosine (Fig. 3 *C* and *D*) to interact with the ATP-binding pocket, similar to the role of the FDDY motif in PKA, as well as in mammalian PKG (32). In the NLT, the hydrophobic motif (HF) features WxxxF in place of FxxF (Fig. 3*C*), a minor deviation conserved in all types of PKG. In our structure, W849 is disordered, but F853 engages a hydrophobic pocket at the top of the N lobe (Fig. 3*D*), similar to what has been reported in PKA (32). In addition, the C tail makes interactions with the N-terminal helix αA_K_ and water-mediated contacts with CNB-D (Fig. 3*E*), the former of which is described below.

The N-terminal helix, αA_K_ in PKG, is the only major structural element to deviate in position noticeably from its PKA counterpart (Fig. 3*F*). As shown in Fig. 3*G*, αA_K_ in *Pf*PKG makes multiple contacts with the N and the C lobes, some of which involve R809 as well as the C terminus of *Pf*PKG–KD, and none of which have been observed in PKA. The contact between the two termini features a salt bridge between R528 and D597 as well as a π-bond between H524 and R809. Substitution with alanine of H524, R528, or R809 (*Pf*PKG–H524A, –R528A, and –R809A; *SI Appendix*, Table S2) resulted in polypeptides that were either unstable (i.e., prone to precipitate) or inactive, establishing the significance of these interactions.

### Structures of the CNB Domains of *Pf*PKG Explain Their Distinct cGMP-Binding Properties.

**Fig. 4.**
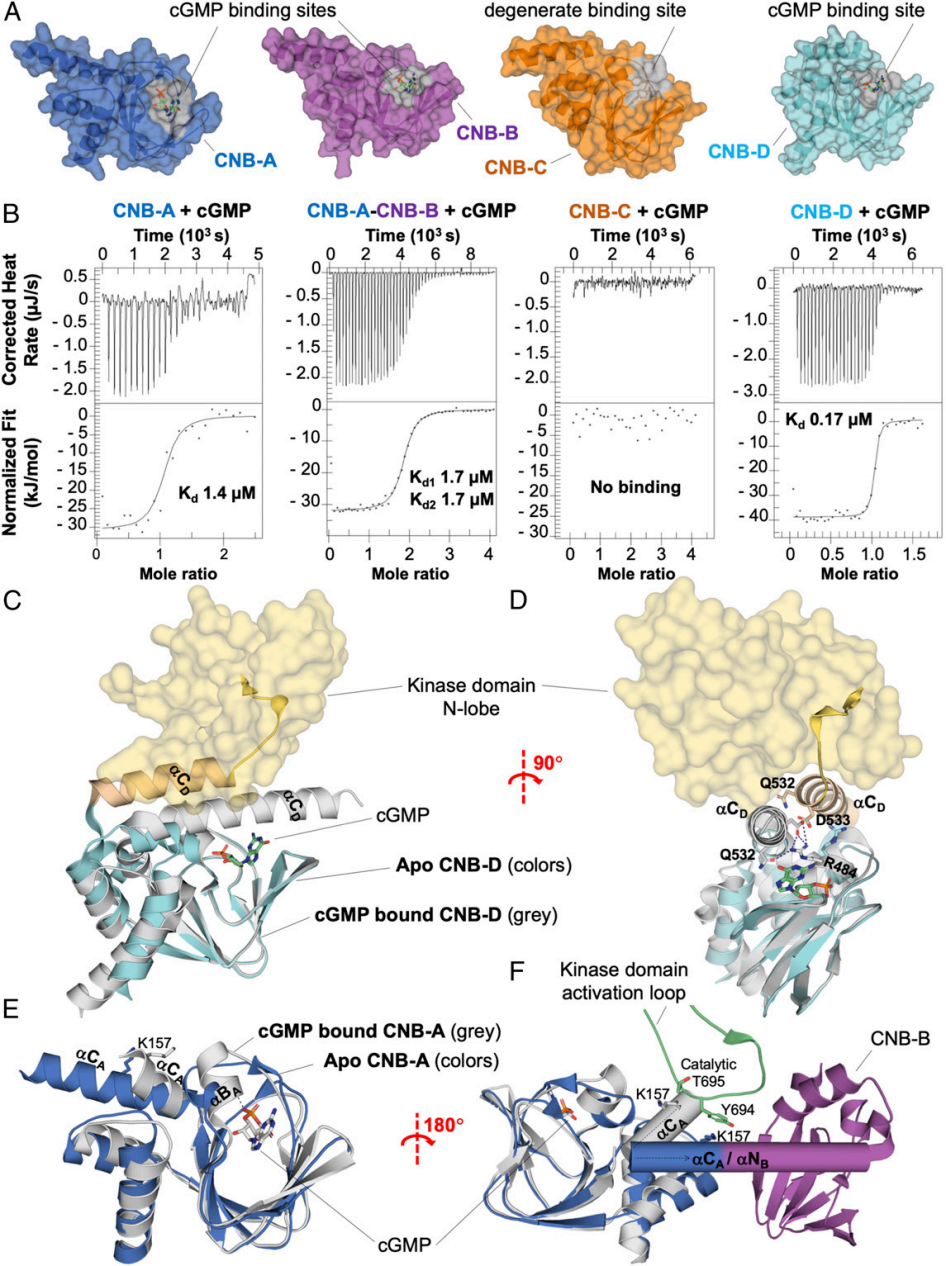
The CNBs of *Pf*PKG. (*A*) Surface rendering of the four *Pf*PKG CNB domains, shown with their cGMP-binding pocket in gray surfaces. A cGMP molecule is manually docked in their putative binding sites for illustration. The third CNB, pCNB-C, is similar overall to the other CNBs, except for the cGMP-binding pocket, which is occluded due to a network of hydrophobic residues. (*B*) ITC binding curves for the CNB-A, -AB, -C, and -D constructs to cGMP. *Upper* and *Lower* display the ITC titration curves and the binding isotherms, respectively. As the CNB-B domain did not yield soluble recombinant protein, its binding activity was tested within a CNB-AB tandem construct. The binding affinity for the CNB-A, -B, and -D domains is 1.4, 1.7, and 0.17 µM, respectively; the CNB-C domain does not bind to cGMP. (*C*) Alignment of the CNB-D in our *apo Pf*PKG structure (in color) with the cGMP-bound costructure (PDB ID code 4OFG; in gray) shows that the helix αC_D_ undergoes noticeable displacement when cGMP is engaged. The triad (R484, Q532, and D533) key to activation of the enzyme is also displaced to (R484′, Q532′, and D533′). (*D*) Orthogonal representation of *C*. (*E*) Alignment of the CNB-A in our *apo Pf*PKG structure (in color) with the cGMP-bound costructure (PDB ID code 5E16; in gray) shows the rearrangement of helices αB_A_ and αC_A_ in response to cGMP binding. Upon cGMP activation, the helix αC_A_ undergoes a large rotation of 42°, but does not contribute to cGMP capping as observed in PKA and *Pf*CNB-D; instead, the cGMP ligand is stabilized by interactions with the backbone of helix αB_A_. (*F*) Alternative representation of *E* that includes the CNB-A neighboring domains of *Pf*PKG (apo form). The cartoon illustration depicts rearrangements of the connecting helix αC_A_ relative to the CNB-B and kinase activation loop, with the disruption of the important K154–Y694 π-bond interaction.

The *Plasmodium* PKG structures contain four distinct CNBs. All four share the reported canonical fold of CNB domains (33, 34), including the N3A bundle (helices αN and αA, with a 3_10_ loop in between) in the N terminus, an eight-stranded β-barrel, followed by a C-terminal hinge made up of helices αB and αC (Fig. 4*A*). In the middle of the β-barrel of CNB-A, -B, and -D is a 24-residue-long phosphate-binding cassette (PBC) featuring the universally conserved glutamate and arginine residues, as well as a short flexible helix, sometimes referred to as the B′-helix (35). Their interactions with cGMP were determined by isothermal titration calorimetry (ITC), revealing 14, 17, and 0.17 µM binding affinities for the CNB-A, -B, and -D when expressed as standalone recombinant protein samples, respectively (Fig. 4*B*).

The third CNB, pCNB-C, stands out because a hydrophobic network consisting of Y363, H373, F371, and F359 occludes the cGMP-binding pocket. Accordingly, we were not able to detect any binding activity toward cGMP due to a degenerate binding site (Fig. 4*B*). Furthermore, D361 and P370 take the places of glutamine and arginine, respectively, which are universally conserved in determined cAMP- and cGMP-binding CNBs (35). Our crystal structures and binding affinities strongly support previous suggestions that only three of the four CNB domains in *Plasmodium* PKG bind cyclic nucleotides (36).

CNB-D stands out from the other regulatory domains in a different way; its C-terminal helices (αB_D_ and αC_D_) are closer to their cGMP-capping positions than in the other CNB domains, as confirmed by comparison with existing structures of cGMP- and cAMP-binding CNBs (*SI Appendix*, Fig. S6). This domain also has the highest affinity for cGMP by two orders of magnitude, compared with CNB-A and -B (Fig. 4*B*), such that this regulatory unit is likely the first domain in *Pf*PKG to become occupied as the cellular concentration of cGMP rises. This is consistent with a model proposed by Kim et al. (23).

### Interdomain Contacts Provide Insight into Regulation of *Plasmodium* PKG by cGMP.

To study the interaction between different domains in the *Plasmodium* PKG pentagon, we divided the structure into two overlapping halves. The left half consists of CNB-A, -B, and the KD. As shown in Fig. 1*D*, this trio of domains is arranged in a way that is highly similar to the PKAR:PKAC heterodimer. Closer examination reveals that some of PKA’s interdomain contacts, previously cataloged into four sites (12, 18), are also conserved in *Plasmodium* PKG, as described immediately below.

In site 1, the basic AIS in *Pf*PKG is docked against the activation loop of the KD in a manner reported for PKA, with key lysines (K15 and K16) replacing homologous arginines (R94 and R95 in PKAR–RIa) in interacting with the P+1 loop on the KD (*SI Appendix*, Fig. S7*A*). Sites 2 and 3 (*SI Appendix*, Fig. S7*B*) are the main interface where CNB-A and -B meet the activation loop, the P+1 loop, and the C lobe of the KD. Key contacts include π-bonds between K157 and Y694 as well as F164 and R692 (the latter supported by a hydrogen bond between N190 and R692). Furthermore, there is a hydrophobic cluster involving the substrate-binding loop (L696), the C lobe (F747), and CNB-A (I127 and H128). These interactions mutually constrain the lower part of the KD and the first cGMP-binding domains similarly to what has been observed in the PKA heterodimer (12, 18, 37) [e.g., the R194^C^–D241^R^–D267^R^ triad in the PKAC/PKAR–RIa heterodimer (18) is replaced by R692:F164:N190 in *Pf*PKG]. To explore the significance of these interactions, we generated mutants of *Pf*PKG and investigated their activity (*SI Appendix*, Table S2). The resulting recombinant proteins of mutants with K157, F164, N190, or Y694 modified to alanine were either unstable or inactive. On the other hand, mutants with H128 and R692 similarly substituted remained stable and active.

In PKA, site 4 is the region of interaction between domain B (specifically helix αB) and the KD (specifically helices αH and αI). This site has been fully described in holoenzymes comprising PKAR–RIa (12) and –RIIa (37). Intriguingly, there is negligible contact in this area observed in the *Plasmodium* PKG structures. This is likely due to the fact that this contact, required in PKA to secure the position of CNB-B, is obviated by the pentagonal single polypeptide arrangement in *Plasmodium* PKG.

In addition to PKA-like interactions, there are parasite-specific interdomain contacts observed in the *Plasmodium* PKG structures, which are represented in the right half of the pentagon (consisting of pCNB-C, CNB-D, and the KD). Notably, αA_K_ is not only the N-terminal helix of the KD, but also the C-terminal αC helix of CNB-D. In this conjoined arrangement, αA_K_ engages CNB-D, particularly its PBC in multiple contacts. This includes a π-bond between R528 and Y417 that is supported by a salt bridge between Q532 and R528 (*SI Appendix*, Fig. S7*C*). In addition, a salt bridge between D533 and R484 (of the PBC of CNB-D) also contributes to the interaction between αA_K_ and the PBC. Not surprisingly, a mutant form of *Pf*PKG with R484 replaced by alanine was enzymatically inactive (*SI Appendix*, Table S2). These interactions, along with other contacts between CNB-D and the C terminus, constitute site 5, which is not found in PKA.

Interestingly, in *Plasmodium* PKG, the junction of CNB-A and -B conserves the long connecting helix observed in PKA (Fig. 1 *C* and *D*). However, between CNB-B and pCNB-C, as well as between pCNB-C and CNB-D (Fig. 1*C*), this helix is twisted, which results in two helices: the C-terminal helix (αC_B_) of the first CNB and the starting helix of the second CNB (αN). In this arrangement, the helix αN makes contact with the preceding CNB, including interactions with the PBC. These connecting helices not only constrain neighboring domains, but are likely also the mediator of interdomain communication, propagating movement in one domain to the other. To further investigate the cGMP allosteric regulation transmitted along connecting helices, we attempted solving the cocrystal structures of other isolated CNB domains bound to cGMP, including CNB-A and -B. The *Pf*CNB-A domain bound to cGMP yielded high-quality crystals diffracting to 1.65-Å resolution (PDB ID code 5E16) and illustrates conformational changes imposed by the repositioning of the helix αB_A_ to lock the cGMP in place within the binding pocket (Fig. 4*E*). This conformational change is accompanied by a larger displacement of the connecting helix αC_A_ (42° rotation) that is anticipated to redraw the neighboring interaction between CNB-A and KD in the full-length protein (Fig. 4*F*). Especially, we denote the probable rearrangement of key interactions upon cGMP activation, particularly the K157–Y694 π-bond interaction (site 2) between the connecting helix αC_A_ and the activation loop in the immediate vicinity of the kinase active site (Fig. 4 *E* and *F* and *SI Appendix*, Table S2). This clearly suggests a mechanism by which conformational modifications induced by binding of cGMP to one CNB could be relayed to adjacent domains.

## Discussion

In mechanistic studies of protein kinases, obtaining their inactive and active structures are important landmarks representing two key states in a regulatory or signaling system. Using a combination of structural biology with biochemical and biophysical assays, our study has established that cGMP-free *Plasmodium* PKG is a monomeric protein held in an autoinhibited state by four main features: (*i*) intrasteric regulation effected by the *cis*-binding of the AIS in the substrate site; (*ii*) immobilization of the two lobes by the CNBs using a number of interdomain contacts; (*iii*) interaction between the N-terminal helix and the C terminus of the KD in a mutually locking arrangement; and (*iv*) arrangement of connecting helices between CNB-B and pCNB-C, pCNB-C and CNB-D, and, most significantly, the conjoined helix between CNB-D and the KD. To the best of our knowledge, the last two features are not only distinct from PKA, but entirely unique among kinases to date.

### Intrasteric Regulation Is Mediated by an N-Terminal AIS.

Autoinhibition in mammalian PKA and PKG involves interactions between the regulatory and catalytic domains (18, 38). The proposed conformational changes mediated by cGMP binding are thought to release PKG from its autoinhibition and allow it to become active. Although the autoinhibitory domains of PKA and PKG do not have a universally conserved sequence, they all share a consensus small residue (most commonly glycine, replaceable by alanine, serine, or valine; underlined in *SI Appendix*, Fig. S7*A*) in the so-called P_0_ position, where a phosphorylation target—namely, serine or threonine—might be found in a pseudosubstrate peptide (e.g., the pseudosubstrate bound in the AIS site in one of the first PKA structures). Alignments of sequences and structures indicate that this residue (Ala18 in *Pf*PKG) is conserved in the AIS of apicomplexan PKGs. This adds weight to the notion that the Plasmodium AIS performs an autoinhibitory function similar to the inhibitory segment (IS) in PKA (18) and AIS in mammalian PKG, particularly in view of the observable increase in basal activity (i.e., in the absence of cGMP) of the truncated version of *Pf*PKG missing this motif.

Although the domain-swapped dimer in our crystal structures suggests the intriguing possibility of an intermolecular regulatory system (in which each protomer extends N-terminal residues to block the substrate-binding site of its dimeric partner), the totality of our biochemical and biophysical data indicates cGMP-free (i.e., inactive) *Plasmodium* PKG to be predominantly a monomer. Accordingly, we were not able to detect *trans*-inhibition between a full-length and a truncated form of *Pf*PKG, as illustrated in Fig. 2*D*. This suggests that the dimeric interaction observed in our crystal structures is likely a crystallographic artifact. Attempts to crystallize *Plasmodium* PKG in a monomeric form have been unsuccessful, allowing us only to speculate on how the AIS might mediate autoinhibition in monomeric PKG-III. We observe that in all available PKA holoenzyme structures, as well as in our *Plasmodium* PKG structures, a short helix follows the inhibitory region and passes over the long helix, connecting the first two CNBs. In comparing the two PKG molecules in the dimer with the PKA structures (Fig. 2*E*), we hypothesize that, as a monomer, this overpass would shift over from position A to position B, thus enabling *Plasmodium* PKG (and possibly other PKG-III enzymes in general) to mediate intrasteric inhibition in the same manner as PKA.

### Interdomain Contacts Regulate the Function of *Plasmodium* PKG.

There are three types of interdomain interactions in *Plasmodium* PKG: (*i*) contacts between the KD, CNB-A, and -B, which are mostly conserved in PKA; (*ii*) contacts between CNB-D and KD (including and particularly the participation of the two termini), which are previously unrecognized and provide insight into how cGMP activation may take place (see below); and (*iii*) arrangement of connecting helices between all neighboring domains except for CNB-A and -B, which suggests a mechanism in interdomain communication in kinases. When some of these interactions were abrogated by cGMP binding at one or more CNBs or by mutagenesis, we observed significant protein aggregation, indicative of their essential role in maintaining the structural integrity and consequently the off state of *Pf*PKG (*SI Appendix*, Table S2).

In PKA, the N-terminal helix may be phosphorylated and myristoylated, with both posttranslational modifications affecting the helical structure of αA_K_ and the protein’s localization to membranes (39). These sites are missing in *Plasmodium* PKG and in mammalian type I PKG [type II PKG has been reported to be myristoylated (40)]. Instead, αA_K_ in *Plasmodium* PKG is a contact interface for the two lobes of the KD, the C terminus and CNB-D, keeping all these domains and subdomains in a mutually constraining arrangement. When cGMP binds CNB-D, at least some of these contacts are expected to be abrogated or rearranged, likely resulting in changes in αA_K_. This hypothesis is confirmed by a study in which Kim et al. (23) identified the formation of a capping triad (R484, Q532, and D533) in CNB-D when cGMP enters its pocket; a comparison of our full-length structure and their cGMP-bound structure (PDB ID code 4OFG) showed that, in order for Q532 and D533 to form this triad and for R484 to engage cGMP, helix αA_K_ has to undergo noticeable displacement (Fig. 4 *C* and *D*). To overcome stability issues of PKG in the presence of cGMP, we produced isolated CNBs to capture cGMP-dependent conformational changes in other CNBs. As such, we report the cocrystallized *Pf*CNB-A structure bound to cGMP, which illustrates the reorganization of helix αC_A_ upon cGMP activation (Fig. 4*E*). The conformational alteration of this connecting helix is expected to make a significant contribution to kinase activity, as it makes important interactions with the kinase activation loop (K157–Y694 π-bond stabilization; Fig. 4*F* and *SI Appendix*, Table S2). The torsion of the connecting helix αC_A_ is reminiscent of cAMP-dependent activation by PKA, which uses a similar mechanism to destabilize the extended αC_A_ helix to uncouple CNB-A and -B before dissociation of the complex (12).

Despite a lack of phosphorylation, the activation loop is in its open (active) conformation. This is presumably necessary to allow docking of the AIS. In kinase structures (including PKA) where the activation loop is phosphorylated, T197 is extended by the addition of phosphate such that it can engage the C-alpha helix, in part to keep the activation loop in the stable conformation. In our structures, the function of the phosphate is instead replaced by interaction with CNB-A (*SI Appendix*, Fig. S7*B*).

### A Structural Relay Model for Activation of *Plasmodium* PKG.

In *Plasmodium* parasites, stringent timing is required for cGMP-mediated events, including egress of merozoites (41) and gametes (4) from erythrocytes. A key trigger in the regulation of this timing is cooperative binding of cGMP and activation of PKG—a property confirmed by the sigmoidal shape of the activity curves of our samples and a Hill coefficient of 1.9 observed in our experiments. Whereas cooperativity in PKA is conferred by the additional constraints imposed in the tetrameric holoenzyme (13), our data indicate that, in *Plasmodium* PKG, and likely PKG-IIIα in general, it is mediated by a network of connecting helices which enables the binding of cGMP at CNB-D to facilitate binding at CNB-B and -A and ultimately removes all inhibitory constraints from the KD. Specifically, we propose the following mechanism of cooperative activation (a sequence that may also represent four potential stable or metastable states of *Pf*PKG). (*i*) When cGMP and ATP concentrations are low, all three functional CNBs and the ATP-binding site are ligand-free. The substrate-binding site of the KD is occupied by the AIS. This is the totally autoinhibited state of *Pf*PKG. (*ii*) As the level of cGMP rises, the domain with the highest affinity, CNB-D, becomes occupied first, resulting in a series of conformational changes that involves displacement of αA_K_. This releases the N lobe of the KD from some of its constraints. *Pf*PKG is only basally active in this state because the AIS continues to inhibit the KD, and the C lobe remains constrained by the first two CNBs. *Pf*PKG (with ATP) with cGMP-bound CNB-D may be a structurally metastable state, one that is primed to engage cGMP at CNB-B. (*iii*) The movement initiated at CNB-D is propagated to pCNB-C via the connecting helix between the domains. In return, this propels movement of the helix shared by CNB-B and pCNB-C, allowing it to be recruited by CNB-B as the capping helix as cGMP moves in, thus initiating bending of the helical bridge between CNB-A and -B in a manner seen in PKA and enabling binding of cGMP to CNB-A. (*iv*) Binding of cGMP in CNB-A disrupts interactions between this domain and the KD, including the hydrophobic stack. This can release the AIS from its autoinhibitory position and the C lobe from all constraints, and free the KD, which is already in its active conformation.

Collectively, our structural-relay model of cooperative activation proposes that cGMP-mediated activation of *Plasmodium* PKG involves a series of conformational changes, initiated by binding to CNB-D, which are propagated around the pentagonal molecule in a relay-like manner, increasing the affinity for cGMP at CNB-B and, ultimately, CNB-A. Binding at three sites leads to a fully active KD in a yet-to-be-determined structural arrangement of the active full-length protein.

## Materials and Methods

### Protein Expression and Purification.

Synthetic DNA for *Pf*PKG and *Pv*PKG from Genscript (sequences are in *SI Appendix*) was amplified by PCR and subcloned into the pFBOH-MHL vector—a baculovirus expression vector with an N-terminal Hexa-Histag followed by a TEV cleavage site (https://www.thesgc.org/reagents/vectors). Individual *Pf*CNB domains (CNB-A, -AB, -C, and -D) were subcloned into the pET15-MHL vector—a bacterial expression vector with an N-terminal Hexa-Histag followed by a TEV cleavage site. *Pf*PKG mutants were cloned by using a described method (42) and expressed and purified as described above.

For protein expression, recombinant viral DNA was generated by transformation of the resulting plasmid into DH10Bac *Escherichia coli* competent cells. This was in turn transferred into Sf9 insect cells by using Cellfectin transfection reagent (Life Technologies, Inc.). The insect cells were grown in HyQ SFX insect serum-free medium (Thermo Fisher Scientific, Inc.), infected with 15 mL of P3 viral stocks per 0.8 L of suspension cell culture, and incubated at 27 °C by using a platform shaker at 100 rpm. The cells were collected at 60- to 72-h postinfection, washed once with phosphate-buffered saline (PBS), and transferred to buffer A (25 mM Hepes, pH 7.5, 500 mM NaCl, 5% glycerol, and 5 mM imidazole). Individual *Pf*CNB domains were produced in *E. coli* BL21 (DE3) grown in Terrific Broth medium supplemented with ampicillin (100 µg/mL) at 37 °C and induced for overexpression at 18 °C with 0.5 mM isopropyl β-d-1-thiogalactopyranoside by using a LEX bubbling system. Cells were incubated for 1 h at room temperature in the presence of 10% 3-[(3-cholamidopropyl)dimethylammonio]-1-propanesulfonate followed by 5 min of sonication by using a dual-horn sonicator (pulse length set to 15 s on and 20 s off). The total cell lysate was centrifuged at 16,000 × *g* for 1 h at 4 °C, and soluble fractions were collected. Protein samples were purified by using affinity chromatography (nickel beads; Sigma). The His-tagged PKG constructs were eluted with buffer A containing 250 mM imidazole. The eluted protein was then subjected to size-exclusion chromatography using an ÄKTAxpress system equipped with a Superdex 200 26/60 column on a system (GE Healthcare) preequilibrated in buffer A.

### Crystallography.

*Pf*PKG, *Pv*PKG, and *Pv*PKG–AMPPNP were set up in sitting-drop vapor-diffusion experiments at room temperature (18 °C). All proteins yielded quality-diffraction crystals in a concentration range of 10–15 mg/mL. *Pf*PKG was crystallized in the C222_1_ space group in 0.4 M l-proline, 10% poly(ethylene glycol) (PEG) 3350, 0.1 M Hepes (pH 7.8), and 15% ethylene glycol. *Pv*PKG was crystallized in the C2 space group in 10% PEG 5000 monomethyl ether, 5% tascimate, 0.1 M Hepes, 15 mM spermidine (pH 7.0), and 25% glycerol. *Pv*PKG–AMPPNP was crystallized in the C2 space group in 18.2% PEG 3350, 0.1 M Hepes (pH 7.0), 0.1 M succinate, 2 mM MgCl_2_, and 5 mM AMPPNP. *Pf*CNB-D was cocrystallized in the C222_1_ space group with 1 mM cGMP in 25% PEG 33350, 0.2 M NaCl, and 0.1 M Hepes (pH 7.5).

For *Pf*PKG (apo form) and *Pv*PKG–AMPPNP (cocrystal), data were collected at beamline 19ID of the Argonne National Laboratory’s Advanced Photon Source (http://www.sbc.anl.gov/index.html) and processed by using HKL-3000 (43). For *Pv*PKG (apo form), data were collected at beamline 23ID and processed by using XDS (44). Finally, the P*f*CNBD–cGMP (cocrystal) data were collected on a home-source X-ray diffractometer (Rigaku FR-E SuperBright rotating anode generator) and processed by using HKL3000 (43). The *Pv*PKG structure was solved by using the molecular-replacement pipeline program BALBES (45). One KD in the asymmetric unit was found by using the program, with additional weak density in the lattice clearly showing additional beta sheets and alpha helices. A C-α backbone trace was made into the weak density. The models for the four CNBs were created by using FFAS03 (46). In an iterative process, the CNB models were manually driven into the backbone traces, and then real-space rigid-body refinement was used in the program Coot (47) to more accurately generate the coordinates. After each CNB was located, a round of REFMAC (48) was run, resulting in improved electron-density maps that enabled placement of the next domain. Loops and side-chain placements not matching the electron density were removed before refinement, and the overall model was built manually by using Coot. The *Pf*PKG structure was solved by using Phaser for molecular replacement and the *Pv*PKG coordinates as a search model. Both *Pf*PKG and *Pv*PKG structures were refined by using REFMAC (49) to final R factors of 20.4% and 21.2%, respectively. The *Pv*PKG–AMPPNP structure was determined by refining the previously determined *Pv*PKG apo structure against the data acquired from the isomorphous complex crystals. Refinement was carried out by using Buster (50) and REFMAC (49) combined with iterative manual model building using the molecular graphics program Coot (47) to a final R factor of 22.1%. The P*f*CNBD–cGMP structure was solved by using Phaser for molecular replacement using the CNB-D domain coordinates from P*f*PKG as a search model; the structure was refined by using REFMAC to a final R factor of 19.6%. The geometry of the final models was checked by using MolProbity (51) for reasonable clash scores and no Ramachandran outliers. Crystallographic details and refinement statistics are summarized in *SI Appendix*, Table S2. All of the illustrations in this work were generated by using MacPymol (Schrödinger, LLC). The coordinates have been deposited in the NCBI protein structure database [https://www.ncbi.nlm.nih.gov/protein; PDB ID codes 5DYL (*Pv*PKG), 5DYK (*Pf*PKG), and 5DZC (*Pv*PKG–AMPPNP)].

### Enzymatic Assays.

Kinase activity was characterized by using an NADH/ATPase coupled assay (52). The reactions were performed at 25 °C in a 384-well plate by using the Synergy 4 plate reader (Biotek). The reaction mix typically contained the enzyme at a concentration of 250 nM, 500 μM Peptide 7 (RRRAPSFYAK), 150 μM NADH, 300 μM phosphoenolpyruvate, 1 mM ATP, a lactate dehydrogenase/pyruvate kinase mix from Sigma (3 units/mL), 20 mM Hepes (pH 7.5), 30 mM NaCl, 10 mM MgCl_2_, 1 mM CaCl_2_, 1 mM dithiothreitol, and 0.01% Tween 20. The reaction was started by adding cGMP (0–200 μM) and monitored for 1 h by using the rate of NADH absorbance decrease at 340 nm, which is proportional to the rate of ATP hydrolysis.

### ITC.

The binding constant and thermodynamic parameters of cGMP binding to the CNB domains of *Pf*PKG were assessed by using a nano-isothermal titration calorimeter (TA Instruments). Experiments with *Pf*CNB-A, -AB, -C, and -D were performed at 25 °C. The sample cell was filled with 169 μL of purified protein samples prepared at a concentration of 60 μM in buffer A [25 mM Hepes, pH 7.5, 300 mM NaCl, and 0.5 mM Tris(2-carboxyethyl)phosphine (TCEP)] and stirred constantly at 350 rpm. The syringe was filled with 50 μL of cGMP at a concentration of 0.7 mM in buffer A and titrated into the sample cell by using 3-μL injections at 180-s intervals. The net binding data were fitted by using the NanoAnalyze Software (TA Instruments) to calculate the binding parameters.

### Analytical Ultracentrifugation.

Sedimentation equilibrium analytical ultracentrifugation experiments were performed by using an Optima XL-A analytical ultracentrifuge (Beckman). Protein samples at concentrations of 0.4, 0.8, and 1.2 mg/mL were prepared in a buffer containing 25 mM Hepes (pH 7.5), 150 mM NaCl, and 1 mM TCEP. The samples were spun for 36 h at 6,000; 8,000; and 10,000 rpm by using an An-60 Ti analytical rotor until equilibrium was reached at 4 °C. Absorbance was monitored at 280 nm. The partial specific volume, solvent density, and solvent viscosity were estimated by using the Sednterp program (University of New Hampshire; server located at http://rasmb.org/sednterp/). Data analysis was done with the Origin MicroCal XL-A/CL-I Data Analysis Software Package (Version 4.0).

### Multiangle Light Scattering.

The molecular size of purified *Pf*PKG was measured at 25 °C by using a Viscotek Tetra detection system equipped with detectors for static light scattering, UV, and refractive index (Malvern Instruments) connected downstream of a size-exclusion chromatography system with a Superdex 200 HR 10/30 column mounted. The column was equilibrated in Equilibrium Buffer (25 mM Hepes, 150 mM NaCl, and 0.5 mM TCEP). Protein samples and the bovine serum albumin standard were dialyzed overnight against Equilibration Buffer. Protein was diluted to a concentration of 6 mg/mL. A volume of 100 μL of both was sequentially injected by using an auto-sampler into the chromatography system at a flow rate of 0.2 mL/min. Molar weight determination was performed by using the Omnisec software (Malvern Instruments).

### *P. falciparum* Culture and Preparation of Soluble Protein Extracts.

Transgenic *P. falciparum* clone 3D7/PfPKG-HA-3A (27) was grown in A+ erythrocytes (National Blood Transfusion Service) according to standard procedures (53) and synchronized by multiple sorbitol treatments (54). Late schizont-stage parasites were released from red blood cells by using 0.15% saponin in PBS and washed twice in PBS. The parasite pellet was resuspended in hypotonic lysis buffer (5 mM Tris⋅HCl, pH 8.0) and frozen at −80 °C. All buffers were supplemented with protease inhibitors (cOmplete protease inhibitor mixture; Roche). The lysate was thawed and centrifuged at 16,000 × *g* for 15 min. The supernatant containing the soluble protein fraction was analyzed by native polyacrylamide gel electrophoresis (PAGE) and immunoblotting.

### Native Gel Electrophoresis and Immunoblotting.

Protein samples were mixed with 2× native sample buffer (62.5 mM Tris⋅HCl, pH 6.8, 25% glycerol, and 1% Bromophenol blue ± DTT) or sodium dodecyl sulfate/PAGE sample buffer + DTT and resolved on an 8% Tris⋅HCl (pH 8.8) polyacrylamide gel run in Tris⋅glycine buffer (pH 8.3) for 3 h at 150 V in an ice/water bath or on an 8% Tris.HCl (pH 8.8) polyacrylamide gel run in Tris.glycine SDS buffer (pH 8.3), respectively. Proteins were transferred to nitrocellulose and the *Pf*PKG-HA fusion protein visualized with anti-hemagglutinin antibody (clone 3F10, Roche; diluted to 1:3,000) followed by horseradish peroxidase-conjugated anti-rat (catalog no. SC-2006, Santa Cruz; at 1:6,000). The blot was reacted with enhanced chemiluminescence plus substrate (Pierce) and exposed to X-ray film.

## Supplementary Material

Supplementary File
