# Supplementary material for: Structures of the cGMP-dependent protein kinase in malaria parasites reveal a unique structural relay mechanism for activation
Source: Proc Natl Acad Sci U S A. 2019 Jun 25;116(28):14164–73. doi: 10.1073/pnas.1905558116 (PMC6628679; doi:10.1073/pnas.1905558116)

PNAS
www.pnas.org

Supplementary Information for

Structures of the cGMP-dependent protein kinase in malaria parasites reveal a novel structural relay mechanism for activation

Majida El Bakkouri, Imène Kouidmi, Amy K. Wernimont, Mehrnaz, Amani, Ashley Hutchinson, Peter Loppnau, Jeong Joo Kim, Christian Flueck, John R. Walker, Alma Seitova, Guillermo Senisterra, Yoshito Kakihara, Choel Kim, Michael J. Blackman, Charles Calmettes, David A. Baker, Raymond Hui

David A. Baker and Charles Calmettes
Email: david.baker@lshtm.ac.uk and charles.calmettes@iaf.inrs.ca

This PDF file includes:

Supplementary text (Appendix I)
Figs. S1 to S7
Tables S1 to S2

www.pnas.org/cgi/doi/10.1073/pnas.1905558116

Appendix I – Synthetic DNA sequences for *Pf*PKG and *Pv*PKG

Using modified coding sequences shown below, we were able to express usable amounts of pure, soluble PKG from *P. falciparum* and *P. vivax* using a previously reported baculovirus system [15].

>*Pf*PKG

ATGGAAGAAGACGATAACCTGAAAAAAGGCAACGAACGCAACAAAAAAAAAGCAATCTTTAGCAACGATGACTTTACGGGCGAAGACAGTCTGATGGAAGA
TCATCTGGAACTGCGTGAAAAACTGTCCGAAGATATTGACATGATCAAAACCAGCCTGAAAAACAATCTGGTGTGCTCTACCCTGAATGATAACGAAATTCTG
ACGCTGTCAAACTACATGCAATTTTTCGTGTTCAAATCGGGCAACCTGGTTATCAAACAGGGTGAAAAAGGCAGTTATTTCTTTATCATCAACTCCGGTAAATT
TGATGTCTACGTGAACGACAAAAAAGTTAAAACCATGGGCAAAGGTAGCTCTTTCGGCGAAGCGGCCCTGATTCACAATACCCAGCGCTCTGCAACGATTATC
GCTGAAACCGATGGTACGCTGTGGGGCGTCCAGCGTAGCACCTTTCGCGCGACGCTGAAACAACTGTCTAACCGTAACTTCAACGAAAACCGCACCTTCATTG
ATAGTGTTTCCGTCTTTGACATGCTGACCGAAGCGCAGAAAAATATGATTACGAACGCCTGTGTGATCCAAAATTTCAAATCTGGCGAAACCATTGTCAAACA
GGGTGATTATGGCGACGTGCTGTACATCCTGAAAGAAGGTAAAGCGACGGTGTATATTAACGATGAAGAAATCCGTGTTCTGGAAAAAGGTAGTTATTTTGGC
GAACGTGCCCTGCTGTACGATGAACCGCGCTCAGCGACCATTATCGCCAAAGAACCGACGGCATGCGCTTCGATTTGTCGTAAACTGCTGAATATCGTGCTGG
GCAACCTGCAGGTGGTTCTGTTTCGCAATATTATGACCGAAGCCCTGCAGCAATCAGAAATCTTTAAACAGTTCTCGGGTGATCAACTGAACGATCTGGCGGAC
ACGGCCATTGTTCGTGACTATCCGGCAAACTACAACATCCTGCATAAAGATAAAGTGAAAAGTGTTAAATACATTATCGTTCTGGAAGGCAAAGTCGAACTGT
TTCTGGATGACACCTCCATTGGTATCCTGAGCCGCGGTATGTCTTTTGGCGATCAGTACGTTCTGAACCAGAAACAACCGTTCAAACACACCATTAAAAGCCTG
GAAGTCTGCAAAATTGCACTGATCACCGAAACGTGCCTGGCTGATTGTCTGGGTAACAATAACATTGATGCGAGTATCGACTACAACAACAAAAAATCCATCA
TTAAGAAAATGTATATTTTTCGTTACCTGACCGACAAACAGTGTAATCTGCTGATCGAAGCCTTCCGTACCACGCGCTATGAAGAAGGCGATTACATTATCCAG
GAAGGTGAAGTGGGCAGCCGCTTCTACATCATCAAAAACGGTGAAGTCGAAATTGTGAAAAACAAAAAACGTCTGCGCACCCTGGGTAAAAACGACTATTTC
GGCGAACGTGCGCTGCTGTATGACGAACCGCGCACGGCCAGTGTTATCTCCAAAGTTAATAACGTCGAATGCTGGTTTGTCGATAAATCCGTGTTCCTGCAGAT
TATCCAAGGCCCGATGCTGGCACATCTGGAAGAACGCATTAAAATGCAGGATACCAAAGTGGAAATGGACGAACTGGAAACGGAACGTATTATCGGCCGCGG
TACCTTTGGTACGGTGAAACTGGTTCATCACAAACCGACCAAAATTCGTTATGCACTGAAATGTGTTTCAAAACGCTCGATCATCAACCTGAACCAGCAAAATA
ACATCAAACTGGAACGTGAAATCACCGCTGAAAATGACCACCCGTTTATTATCCGTCTGGTGCGCACGTTCAAAGATTCAAAATACTTCTACTTCCTGACCGAA
CTGGTTACGGGCGGTGAACTGTATGATGCCATTCGCAAACTGGGCCTGCTGTCAAAATCGCAGGCACAATTTTACCTGGGTTCGATTATCCTGGCTATTGAATA
TCTGCATGAACGTAATATCGTCTACCGCGATCTGAAACCGGAAAACATTCTGCTGGACAAACAAGGCTATGTGAAACTGATCGATTTCGGTTGCGCGAAAAAA
GTTCAGGGCCGTGCTTATACCCTGGTCGGTACGCCGCACTACATGGCACCGGAAGTCATTCTGGGCAAAGGTTATGGCTGTACCGTGGATATTTGGGCTCTGGG
CATCTGCCTGTACGAATTTATCTGTGGTCCGCTGCCGTTCGGCAACGATGAAGAAGACCAACTGGAAATTTTTCGCGACATCCTGACCGGTCAGCTGACGTTCC
CGGATTATGTGACCGATACGGACAGCATTAATCTGATGAAACGTCTGCTGTGCCGCCTGCCGCAGGGTCGTATTGGCTGTAGCATCAACGGCTTCAAAGATATT
AAAGACCATCCGTTTTTCTCTAACTTCAACTGGGATAAACTGGCAGGTCGCCTGCTGGACCCGCCGCTGGTTAGCAAATCTGAAACCTATGCCGAAGATATTGA
CATCAAACAGATTGAAGAAGAAGACGCAGAAGACGATGAAGAACCGCTGAACGACGAAGACAACTGGGACATTGATTTT

>*Pv*PKG

ATGCGCTGTAACGAACGTAACAAAAAAAAAGCAATCTTCTCGAATGACGATTTCTCGGGCGAAGACACCCTGATGGAAGACCACCTGCAGCTGCGTGAAAAA
CTGTCCGAAGACATTGAAATGATCAAAGCATCCCTGAAAAACAATCTGGTGTGCTCAACCCTGAACGATAATGAAATCCTGACGCTGTCAAACTACATGCAAT
TTTTCGTGTTCAAAGGCGGTGATCTGGTTATTAAACAGGGTGAAAAAGGCTCGTATTTCTTTATCATCAACAGCGGCAAATTCGATGTCTACGTGAACGACAAA
AAAGTGAAAAGCATGGGCAAAGGTAGCTCTTTCGGTGAAGCGGCCCTGATTCATAATACCCAGCGCTCTGCAACGATCATGGCTGAAACCGATGGTACGCTGT
GGGGCGTTCAGCGTAGTACCTTTCGCGCCACGCTGAAACAACTGTCTAACCGTAACTTCAACGAAAACCGCAGTTTCATCGATAGTGTTTCCGTCTTTGACATG
CTGACCGAAGCACAGAAAAACATGATTACGAATGCTTGTGTGATCCAAATGTTCAAACCGGGTGAAACCATCGTTAAACAGGGTGATTATGGCGACGTCCTGT
TTATTCTGAAAGAAGGTAAAGCAACCGTGTTCATTAACGATAAAGAAATCCGTGTTCTGAATAAAGGTTCCTATTTTGGCGAACGTGCTCTGCTGTACGATGAA
CCGCGCTCGGCGACCATTATCGCCAAAGAACCGACGGCATGCGCTAGCATTTGTCGCAAACTGCTGAACATCGTGCTGGGCAATCTGCAGGTGGTTCTGTTTCG
TAACATTATGACCGAAGCACTGCAGCAATCTGAAATCTTTCGCCAGTTCAGTGCTGAACAACTGAACGATCTGGCGGACACCGCCATTGTTCGTGACTATCCGG
CGAATTACCATATCCTGCACAAAGATAAAGTCAAATCTGTGAAATACCTGATTGTCCTGGAAGGCAAAGTGGAACTGTTTCTGGATGACGAATCCATTGGTAT
CCTGACCCGTGGTAAATCATTTGGCGACCAGTACGTGCTGAATCAGAAACAAAAATTCCGCCATACCGTTAAATCCCTGGATGTCTGCAAAATTGCGCTGATCA
CGGAATCATGCCTGGCCGATTGTCTGGGCGACAACAATATTGATGCATCGATCGACCACAACAACAAAAAATCTATCATTAAGAAAATGTACATCTTTCGTTA
CCTGTCGGAACAGCAATGTAACCTGCTGATTGAAGCGTTTCGTACCACGCGCTATGAAGAAGGTGATTATATTATCCAGGAAGGTGAAGTTGGCTCTCGCTTTT
ATATTATCAAAAACGGCGAAGTGGAAGTTACCAAAAATGGTAAACGTCTGCGCACGCTGGGTAAAAATGACTATTTCGGCGAACGTGCGCTGCTGTATGACGA
ACCGCGCACCGCATCAATTATCTCGAAAGCTACGTCGGTCGAATGCTGGTTTGTGGATAAAAGCGTTTTCCTGCAGATTATCCAAGGCCCGATGCTGACCCATC
TGGAAGAACGCATTAAAATGCAGGATACCAAAGTTGAAATGCACGAACTGGAAACGGAACGTATTATCGGCCGCGGTACCTTTGGTACGGTTAAACTGGTCCA
TCACAAACCGACCCAGATTCGTTATGCGCTGAAATGCGTGAGCAAACGCTCTATTATCAGTCTGAACCAGCAAAACAACATCAAACTGGAACGTGAAATCACC
GCCGAAAATGACCATCCGTTTATTATCCGTCTGGTTCGCACGTTCAAAGATAGCAACTGTTTTTATTTCCTGACCGAACTGGTCACGGGCGGTGAACTGTACGA
TGCCATTCGCAAACTGGGCCTGCTGTCTAAACCGCAGGCACAATTTTATCTGGGTAGTATTATCCTGGCTATTGAATATCTGCATGAACGTAACATCGTGTACC
GCGATCTGAAACCGGAAAATATTCTGCTGGACAAACAAGGTTACGTTAAACTGATTGATTTCGGTTGCGCGAAGAAAATTCAGGGCCGTGCCTATACCCTGGT
CGGTACGCCGCACTACATGGCGCCGGAAGTGATTCTGGGCAAAGGTTATGGCTGTACCGTGGATATTTGGGCCCTGGGCGTGTGCCTGTACGAATTTATTTGTG
GTCCGCTGCCGTTCGGCAATGATCAGGAAGACCAACTGGAAATTTTTCGCGACATCCTGACCGGTCAGCTGACGTTCCCGGATTATGTGTCCGATCAAGACTCA
ATCAACCTGATGAAACGTCTGCTGTGCCGCCTGCCGCAGGGTCGTATTGGCTGTAGTATCAACGGCTTCAAAGACATTAAAGAACACGCGTTTTTCGGCAACTT
CAATTGGGATAAACTGGCAGGTCGCCTGCTGGAACCGCCGCTGGTTAGCAAAGGTGAAACCTACGCGGAAGATATTGACATCAAACAGATTGAAGAAGAAGA
TGCTCTGAACGAAGGTGAACCGCTGGATGGTGACGATAGTTGGGATGTTGACTTTTGA

Appendix II – Supplementary Figures

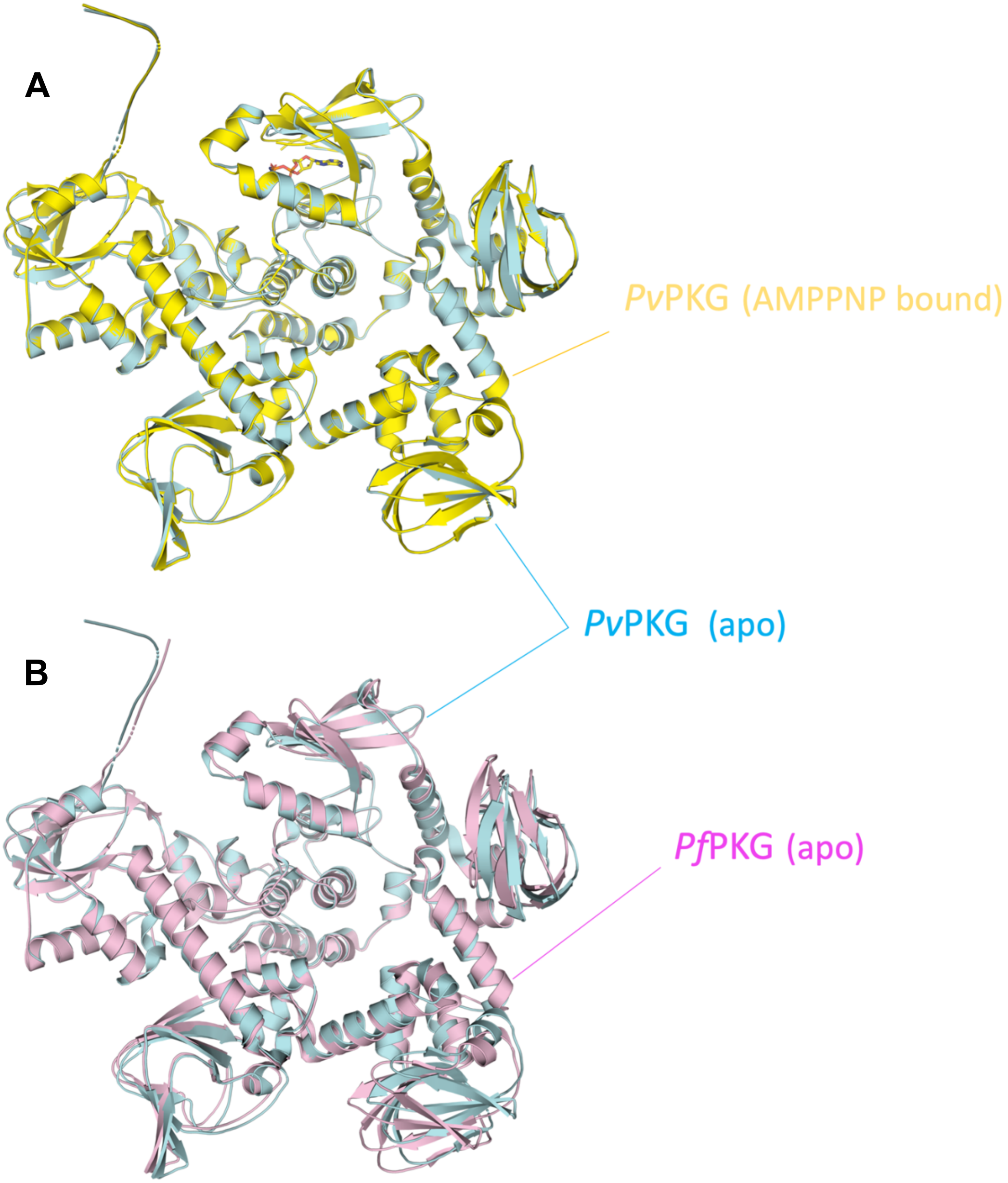

Fig. S1. Structural overlay of PKG structures. Panel A presents the overlaid crystal structures of *P. vivax* PKG, comparing the apo state (cyan) and the holo configuration bound to AMPPNP (yellow). The structural alignment yields an RMSD of 0.2 Å representing negligible differences. Panel B depicts the structural conservation between homologous PKG crystallized in apo states from *P. vivax* (cyan) and *P. Falciparum* (pink) parasites, measuring an RMSD of 0.8 Å – representing negligible differences.

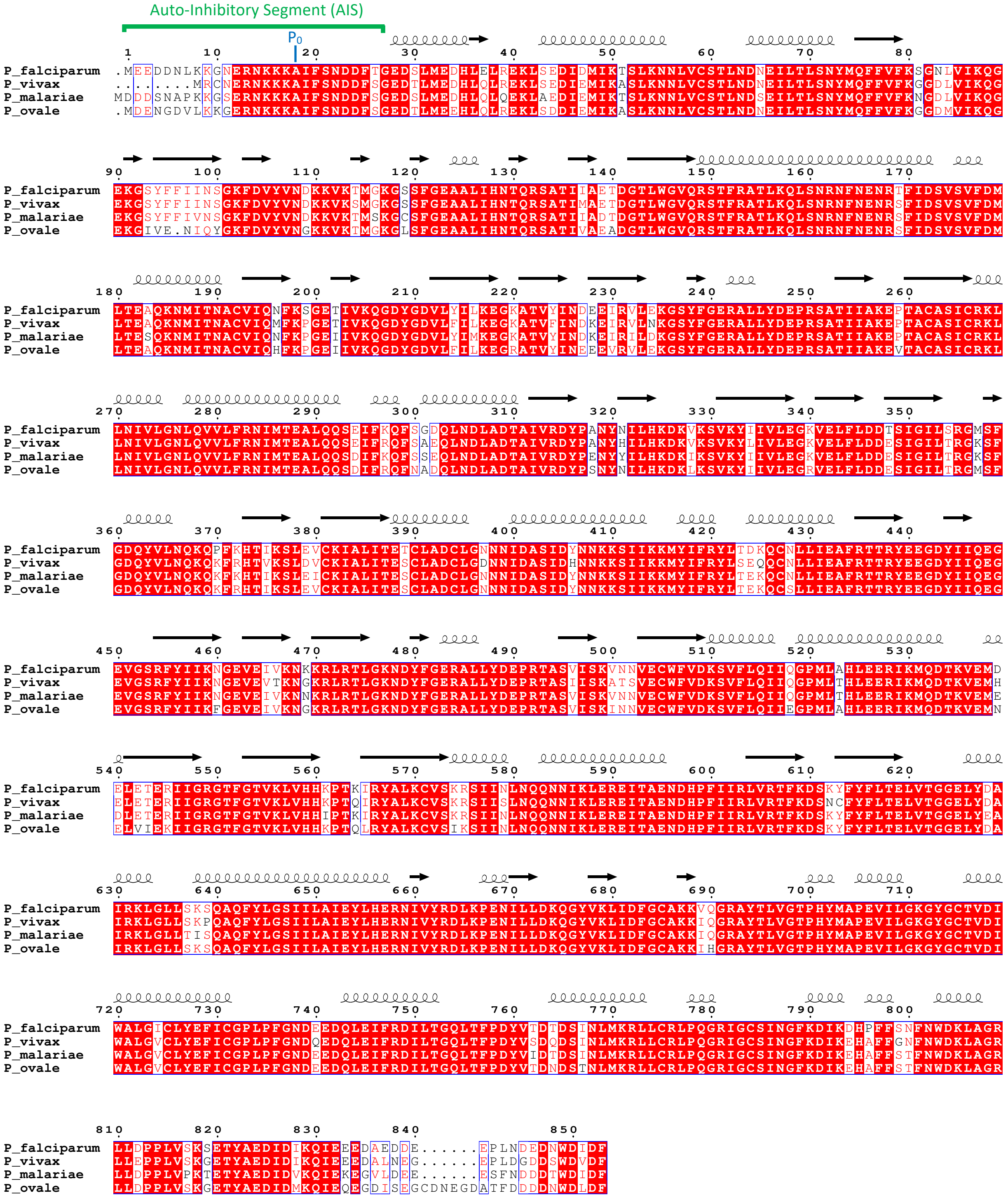

Fig. S2. PKG is conserved in plasmodium parasites. The sequence alignment denotes the structural and sequence conservation of PKG among plasmodium parasites, comparing *P. falciparum*, *P. vivax*, *P. malariae* and *P. ovale*. Identical residues are highlighted in red, and conserved resides are depicted in blue boxes. The secondary structure elements (β-strands and α-helices) from *P. falciparum* are shown above the sequences. The AIS is indicated with a green bar, and the alanine 18 positioned at the P_0 site within the kinase catalytic site is marked in blue.

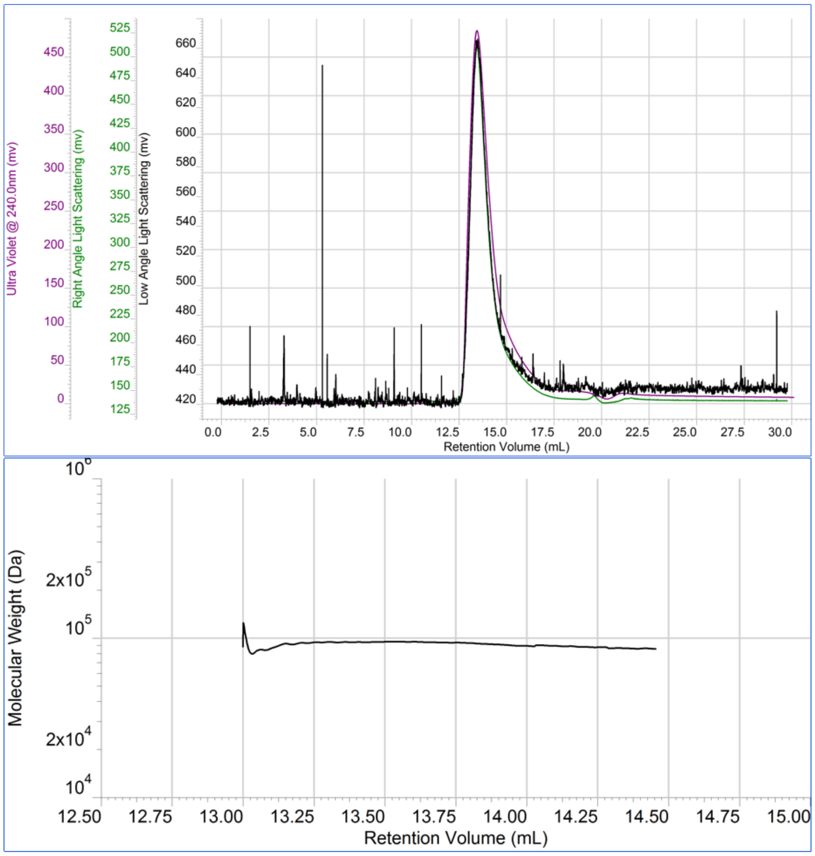

Fig. S3. *Pf*PKG is monomeric in solution. The oligomerization state of the full length *Pf*PKG at 6 mg/ml was assessed using multi-angle laser light scattering (MALLS). The single peak corresponds to the monomeric form of *Pf*PKG eluting at 13.43 mL. The number average molecular weight (Mn) among the PKG elution peak is 91.6 kDa, while the average molecular weight (Mw) is calculated to be 91.9 kDa, indicating that the sample is monomeric in solution (theoretical mass of 98 kDa). The polydispersity index (ratio Mw/Mn) of 1 indicates that PKG is eluted as a monodispersed species.

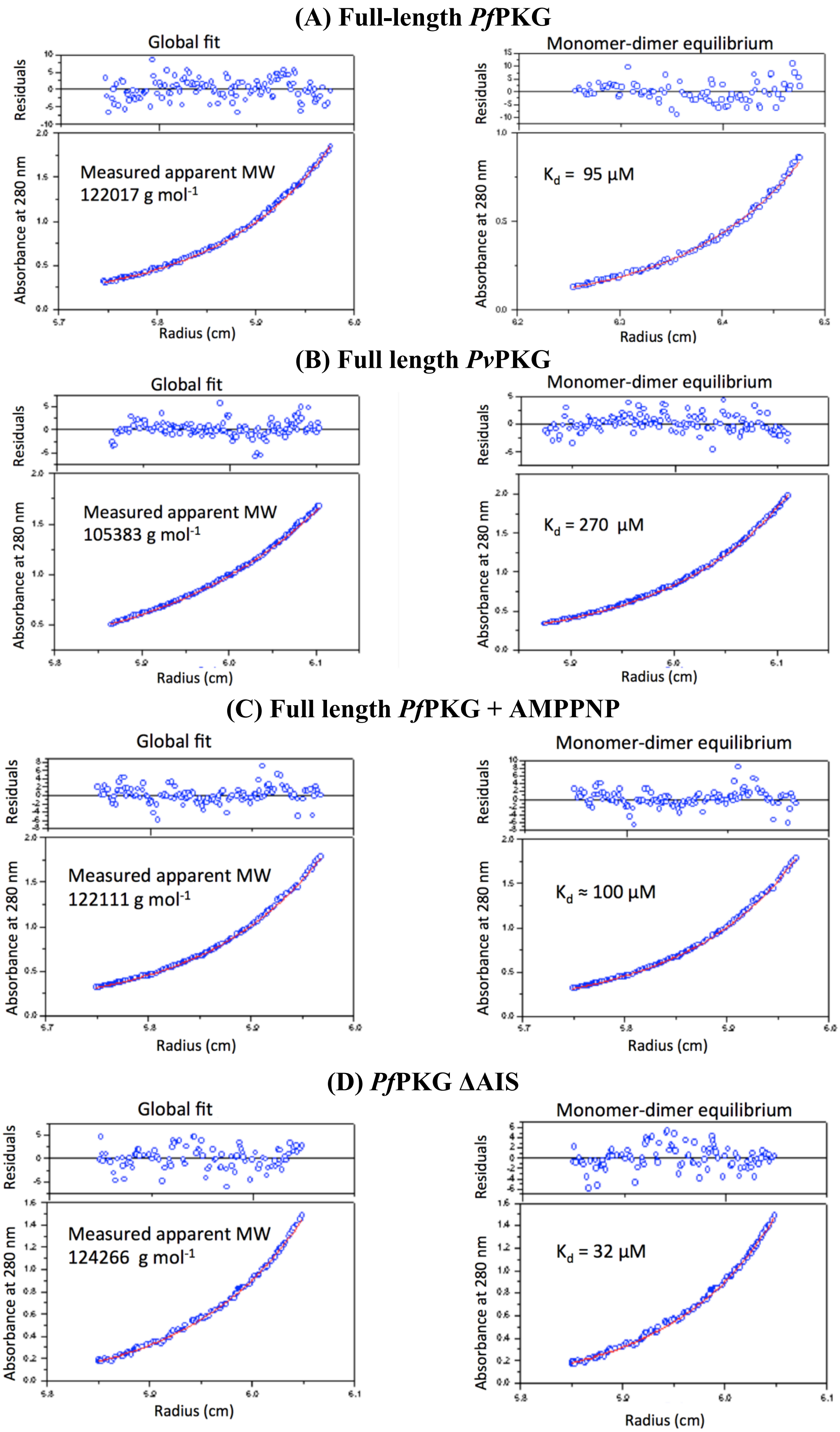

Fig. S4. PKG is monomeric in solution. Data from analytical ultracentrifugation sedimentation equilibrium experiments are shown for four samples each performed at various concentrations (0.4, 0.8

and 1.2 mg/mL): full-length *Pf*PKG (**A**); full-length *Pv*PKG (**B**); full-length *Pf*PKG in the presence of 100 µM AMPPNP (**C**); *Pf*PKG ΔAIS - truncation of the autoinhibitory segment (**D**). The collected data were fitted for global self-association (the average molecular weight deduced and indicated in each case) as well as for monomer-Dimer model (the dissociation constant was deduced and indicated in each case). The 4 constructs, *Pf*PKG, *Pv*PKG, *Pf*PKG bound to AMPPNP, and *Pf*PKG ΔAIS (truncation of the autoinhibitory segment) are monomeric in solution with a calculated apparent molecular weight of 100 to 120 kDa (theoretical mass of 98kDa). The high K_d values suggest the potential formation of homodimers at very high protein concentrations above 10 mg/ml (non-physiological condition).

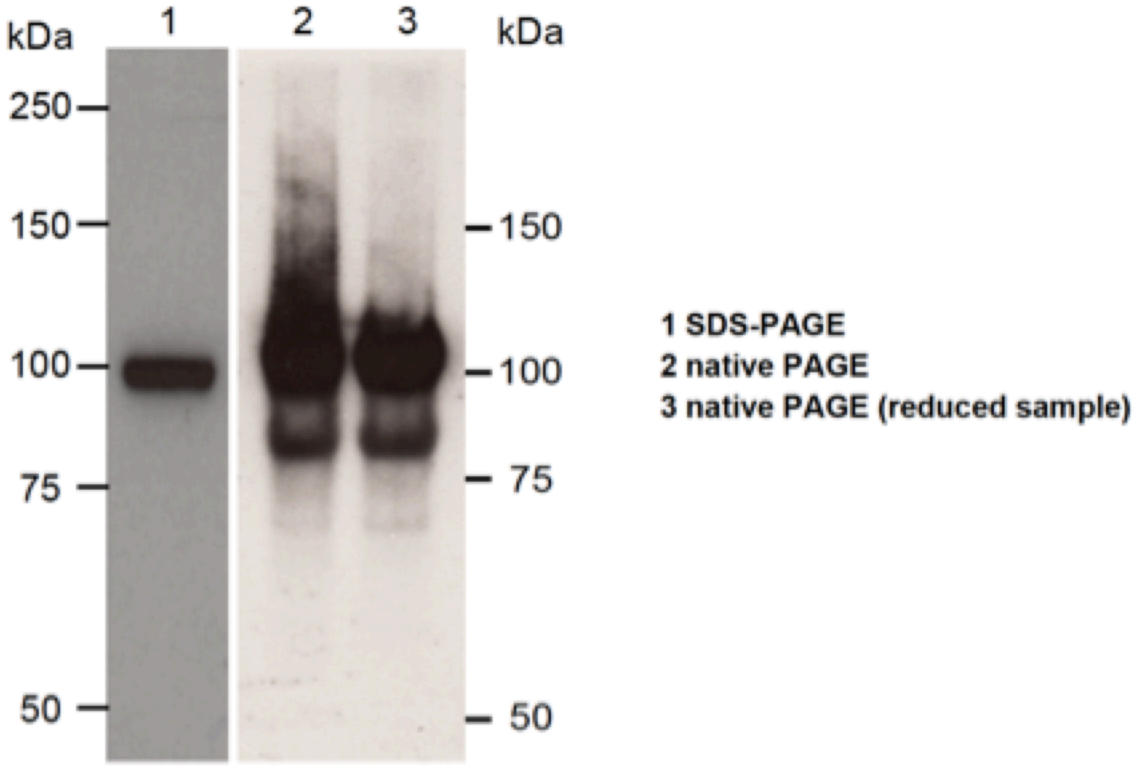

Fig. S5. *Pf*PKG is monomeric *in vivo*. Western blot of *Pf*PKG extracted from schizonts – No evidence of dimer (200+ kDa) at all. Cytosolic extract from 3D7 wild-type parasites resolved on SDS-PAGE and reacted with an anti-human PKG antibody (lane 1). Cytosolic extract from 3D7/PKG-HA parasites resolved on native PAGE and reacted with anti-HA antibody (lanes 2 and 3).

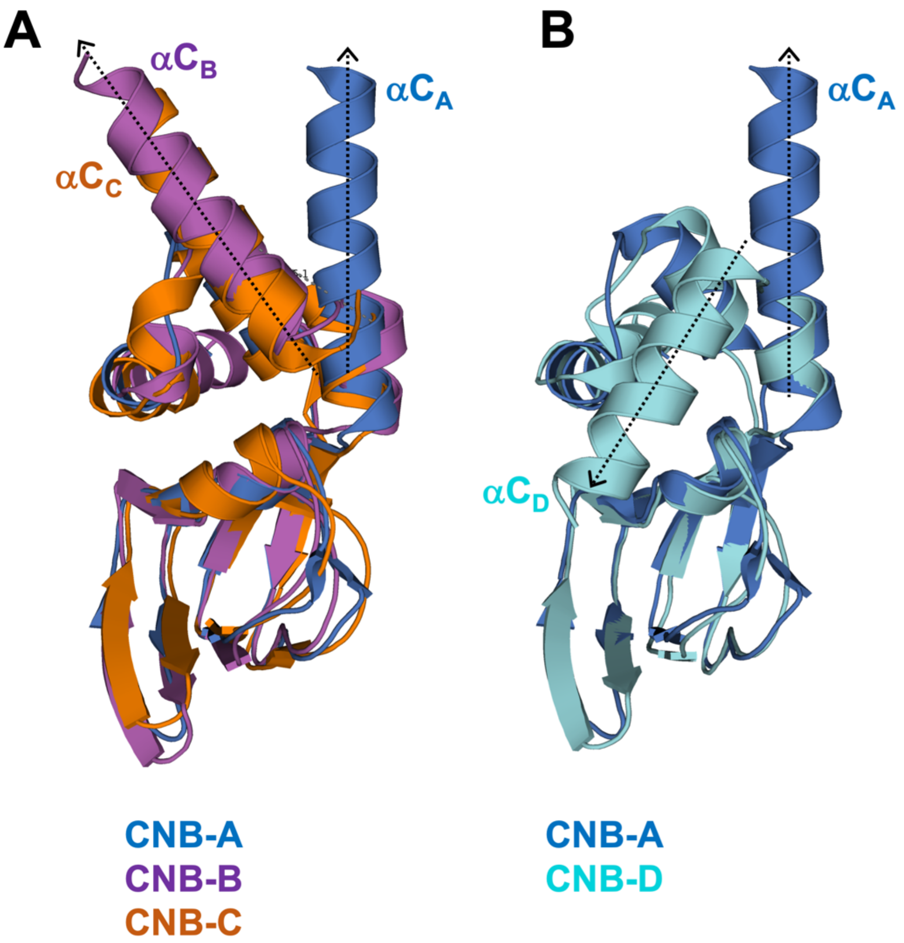

Fig. S6. Cartoon representation of CNBs. The panel **A** depicts a structural overlay of CNB-A, CNB-B and CNB-C. The figure clearly denotes deviations in the direction of helices α_C, which is more open in CNB-A (in blue) than in the other two CNBs. In panel **B**, CNB-A and CNB-D are superposed on each other, showing that α_C in CNB-D is closed in comparison. In fact, with the capping helix in its closed position, CNB-D is closest to its cGMP-bound conformation of all four CNBs, despite the absence of cGMP in the structure.

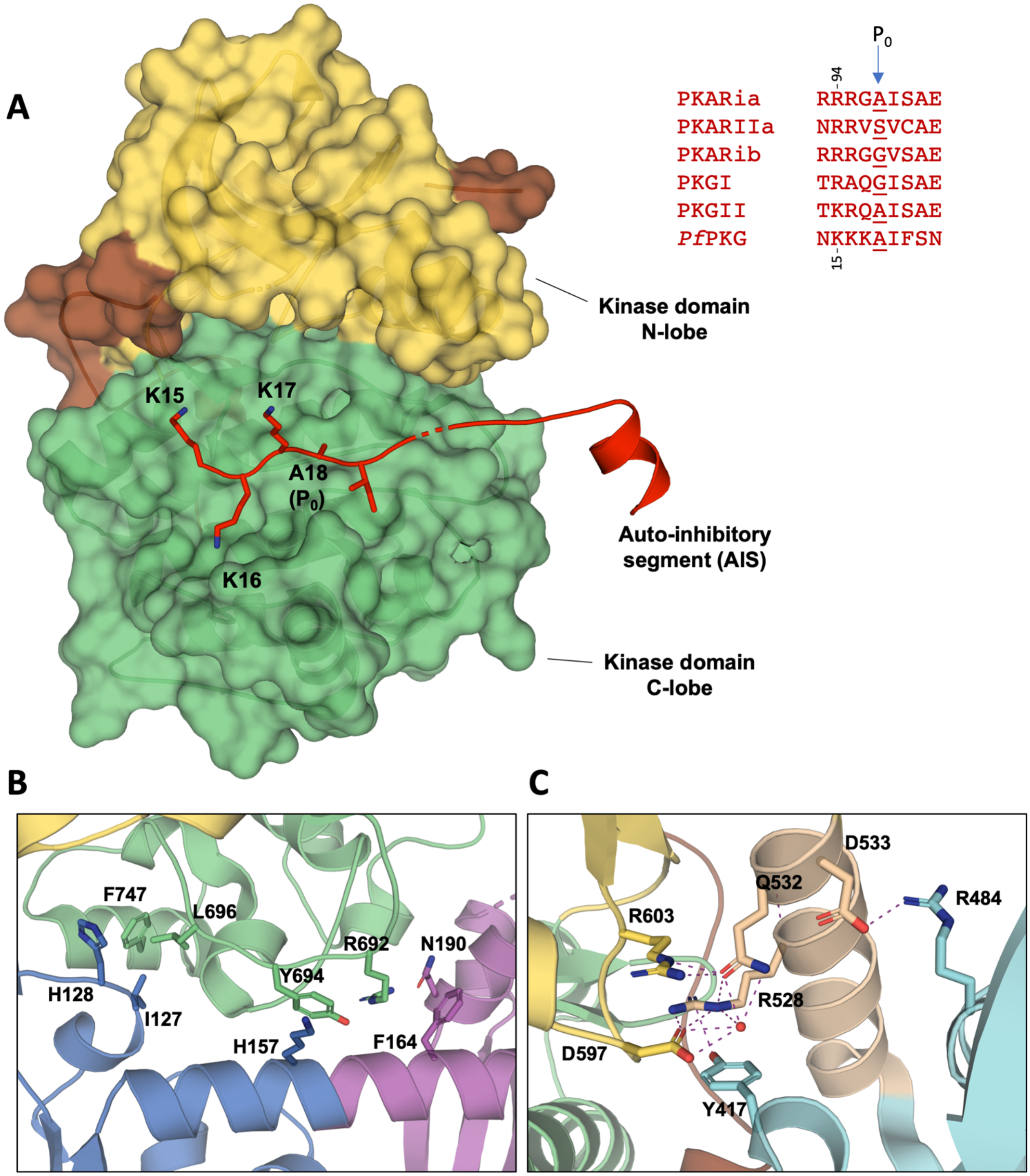

Fig. S7. **Inter-domain contacts conserved in PKA.** Panel **A** is a close-up view of site 1. Here, the AIS is docked against the activation loop of the KD in a PKA-like manner, with K15 and K16 replacing R94 and R95 in PKAR-RIa in interacting with the P+1 loop on the KD. Panel **B** depicts sites 2 and 3. Here, the C-lobe of the KD, including the activation segment, interacts with CNB-A and CNB-B at multiple contacts for mutual constraint and stabilization. These contacts include a hydrophobic cluster involving I19 on the AIS, I127 and H128 on CNB-A and L696 and Y747 on the KD. A pair of π-bonds, namely

K157:Y694 and F164:692 (buttressed by a salt bridge between N190 and R692) are the other notable interactions. Panel **C** describes interdomain contacts between the CNB-D and the KD. Helix α_K conjoins CNB-D and the kinase domain when *Plasmodium* PKG is cGMP-free. This arrangement is held together by a network of interactions that include a π-bond between R528 and Y417 (supported by a salt bridge between R528 and Q532), a salt bridge between D533 and R484, another between R603 and Q532, as well as water-mediated contacts between Q532, R528 and D597.

Table S1 – Crystallographic data

	*Pf*PKG	*Pv*PKG	*Pv*PKG + AMPPNP	*Pf*CNB-A + cGMP
**PDB ID**	5DYK	5DYL	5DZC	5E16
**Data Collection**				
Cell Dimensions	$C222_1$	C2	C2	$C222_1$
Space Group:				
a (Å)	94.51	192.47	193.11	51.27
b (Å)	127.28	117.77	117.91	53.85
c (Å)	215.10	67.68	67.73	92.35
α (°)	90.00	90.00	90.00	90.00
β (°)	90.00	94.66	95.08	90.00
γ (°)	90.00	90.00	90.00	90.00
Wavelength	0.97918	0.97945	0.97929	1.54
Resolution	40.0 - 2.45 (2.49)	45.0 - 2.4 (2.47)	50.0-2.3 (2.38)	50.0-1.65 (1.68)
R_{sym}	0.093 (0.935)	0.085 (0.760)	0.09 (0.845)	0.043 (0.303)
I/σI	8.3 (1.8)	7.7 (1.5)	16.8 (1.2)	
Completeness (%)	96.6 (93.1)	99.9 (99.9)	99.1 (95.5)	99.7 (96.8)
Redundancy	4.7 (4.4)	3.4 (3.4)	4.1 (3.7)	7.4 (5.6)
**Refinement**				
Resolution (Å)	40.0 - 2.45	45 - 2.40	50.0-2.3	50.0-1.65
Number of Reflections	44920	58746	62646	15745
R_{work}/R_{free}	0.204 / 0.240	0.212 / 0.246	0.221/0.254	0.196/0.227
Number of Atoms				
Protein	6468	6294	6437	1078
Water	171	124	144	104
AMPPNP			31	
cGMP				23
Average B-factors	51.7	74.70	74.39	27.09
RMS deviations				
Bond lengths (Å)	0.007	0.007	0.01	0.013
Bond Angles (°)	1.15	1.08	1.36	159.2
Ramachandran plot				
Favored regions	96.6 %	97.8 %	96.5 %	96.8%
Allowed regions	99.8 %	99.9 %	100 %	100%

Table S2 – list of mutations or truncations, and behavior of resulting mutants

Mutation	Interaction or motif affected	Result and relevance
N-terminal truncation	Auto-inhibitory segment	With cGMP, this construct was as active as the full-length recombinant sample. Without cGMP, this construct demonstrated a low but detectable level of activity. In contrast, the full-length sample was inactive without cGMP.
R528A	R528-D597<br>R528-Q532<br>R528-Y417	Mutant sample was unstable and precipitated so much during concentration, leaving insufficient soluble protein for assays. R528 is involved in at least 3 interactions between CNB-D, the C-Lobe and the C-tail. Instability of the mutant suggests that these interactions are key in stabilizing the auto-inhibited configuration of *Pf*PKG
Y417A	R528-Y417	Mutant sample was unstable. Although a low-concentration sample could be collected for assays, it was found inactive.
R809A	H524-R809	Mutant sample was unstable. Although a low-concentration sample could be collected for assays, it was found inactive. Residues H524, R809 along with R288, Q532 and Y17 are all involved in a network of interactions that seems to be crucial for the auto-inhibited form integrity
H524A	H524-R809	Same as above.
R484A	R484-D533	Mutant sample was inactive. R484 is essential for cGMP binding to CNB-D. In its absence, *Pf*PKG could not effectively bind cGMP at this site.
R692A	R692-F164<br>R692-N190	Mutant sample was stable and fully active. This suggests R692 is not essential for PKG activity.
F164A	F164-R692	Mutant sample yielded a very low level of soluble protein, which was found inactive.
N190A	N190-R692	Mutant sample yielded a very low level of soluble protein, which was found inactive.
Y694A	Y694-K157	Mutant sample yielded a very low level of soluble protein, which was found inactive. The Y694-K157 connects the C-lobe to the regulatory domain, and is essential for overall structural integrity.
K157A	Y694-K157	Mutant sample did not yield a usable amount of soluble protein. The Y694-K157 connects the C-lobe to the regulatory domain, and is essential for overall structural integrity.
E589A	ATP-binding site	Mutant sample was stable but inactive. E589 is required for catalytic activity.

The localization of the listed mutations is illustrated in the graphic representation

Table S2 - graphic representation. A map of key inter-domain contacts shows the outcome of mutagenesis experiments listed in table S2. The mutated residues are drawn using color sphere representation. Color code: Red – mutagenesis led to loss of protein expression. Yellow – mutagenesis led to loss of activity. Green – mutagenesis did not change expression or enzymatic behavior of protein.

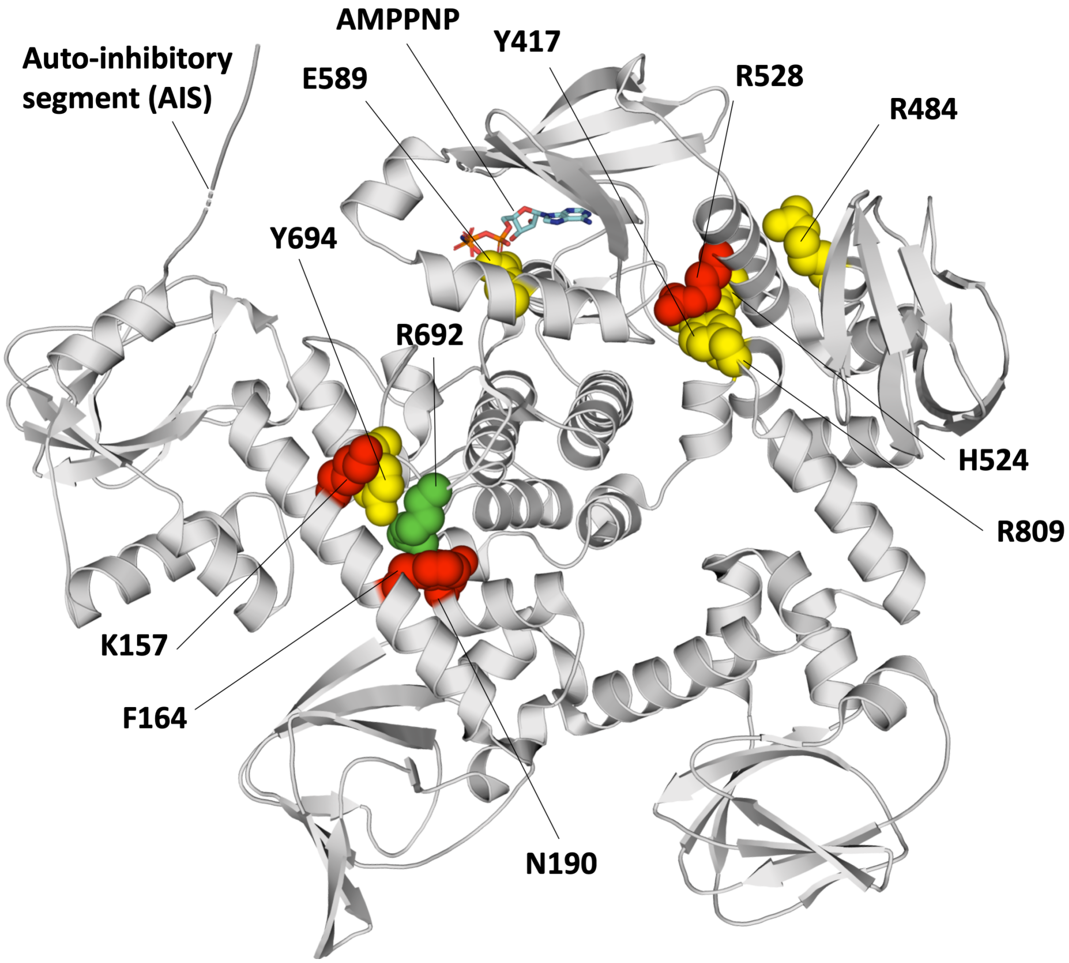

Supplement: Supplementary File [file pnas.1905558116.sapp.pdf]
